# From Waste to Beauty: Agri-Food By-Products as Sources of Antioxidant Compounds for Cosmeceutical Applications

**DOI:** 10.3390/antiox15080949

**Published:** 2026-07-30

**Authors:** Alessia Silla, Maria Cristina Barbalace, Cristiana Caliceti, Angela Punzo, Silvana Hrelia, Cristina Angeloni, Marco Malaguti

**Affiliations:** 1 Department of Biomedical and Neuromotor Sciences, Alma Mater Studiorum, University of Bologna, 40126 Bologna, Italy; cristiana.caliceti@unibo.it (C.C.); angela.punzo2@unibo.it (A.P.); 2 Biostructures and Biosystems National Institute (INBB), 00165 Rome, Italy; 3 Department for Life Quality Studies, Alma Mater Studiorum, University of Bologna, 47921 Rimini, Italy; silvana.hrelia@unibo.it (S.H.); cristina.angeloni@unibo.it (C.A.); marco.malaguti@unibo.it (M.M.); 4 Interdepartmental Centre for Renewable Sources, Environment, Sea and Energy (CIRI FRAME), Alma Mater Studiorum, University of Bologna, 40126 Bologna, Italy; 5 Interdepartmental Centre for Industrial Agrofood Research-CIRI Agrofood, University of Bologna, 47521 Cesena, Italy

**Keywords:** agri-food by-products, cosmeceuticals, antioxidants, skin care, oral care, hair care, green extractions

## Abstract

Agri-food processing generates large quantities of residues that are increasingly recognized as sustainable sources of bioactive ingredients for high-value applications. This narrative review examines agri-food by-products as sources of antioxidant compounds and provides an integrated mechanistic perspective on their applications in skin, oral, and hair care. By highlighting the shared antioxidant mechanisms of action underlying these applications, the review offers a comprehensive framework for understanding the cosmeceutical potential of agri-food-derived bioactive compounds. Peer-reviewed studies published mainly between 2015 and 2026 were considered, focusing on the chemical composition of by-products, green recovery strategies, biological mechanisms, and evidence from in vitro, ex vivo, in vivo, and clinical studies. Fruit and vegetable peels, seeds, skins, pomaces, and other agro-industrial residues contain polyphenols, flavonoids, phenolic acids, tannins, carotenoids, vitamins, lipids, peptides, and polysaccharides with antioxidant, anti-inflammatory, antimicrobial, photoprotective, anti-aging, moisturizing, and pigmentation-modulating properties. These compounds can reduce oxidative stress, support endogenous antioxidant defences, modulate redox-sensitive signaling pathways, attenuate inflammation, preserve extracellular matrix integrity, and improve skin barrier-related outcomes. Green extraction technologies, including ultrasound-assisted extraction, microwave-assisted extraction, supercritical fluid extraction, pressurized liquid extraction, and natural deep eutectic solvents, enhance their sustainable recovery. Overall, agri-food by-products represent promising resources for circular cosmeceutical innovation, although extract standardization, safety, stability, skin bioavailability, formulation performance, and controlled clinical validation remain essential for translation.

## 1. Introduction

The increasing demand for food products, coupled with the expansion of food processing activities, has led to the generation of substantial quantities of agricultural and food-processing residues. It is estimated that nearly one-third of the food produced globally for human consumption is lost or wasted along the supply chain, resulting in significant economic losses and severe environmental consequences [1]. Food waste contributes to the depletion of natural resources, including water, energy, and arable land, while also accounting for a considerable proportion of global greenhouse gas emissions [2]. Consequently, the sustainable management of agri-food waste has emerged as a major challenge for governments, industries, and researchers seeking to develop more resilient and environmentally responsible production systems.

Agri-food by-products are generated throughout the entire food value chain, from agricultural production and harvesting to industrial processing and distribution [3]. These residues include fruit and vegetable peels, seeds, pomaces, stems, leaves, cereal bran, oilseed cakes, dairy by-products, etc. Traditionally, such materials have been regarded as low-value waste streams, frequently failing to exploit their significant biological and economic potential. In recent years, growing environmental concerns and the transition toward more sustainable production models have stimulated a paradigm shift in the perception of agri-food residues, transforming them from waste materials into valuable renewable resources [4,5,6].

This transition is closely associated with the principles of the circular economy and circular bioeconomy, which promote the efficient use of resources through waste minimization, material recovery, and value creation [7,8]. Within this framework, agri-food by-products are increasingly recognized as sustainable materials for the recovery of bioactive compounds that can be employed in high-value applications in pharmaceuticals, cosmetics, and functional foods. The valorization of food-processing residues not only reduces environmental burdens associated with waste disposal but also generates new economic opportunities by converting underutilized biomass into functional ingredients, thereby extending the lifecycle of biological resources.

The relevance of this approach is further highlighted by its alignment with several Sustainable Development Goals (SDGs) established by the United Nations 2030 Agenda [9,10]. In particular, the valorisation of agri-food by-products contributes directly to SDG 12 (Responsible Consumption and Production) by promoting resource efficiency and waste reduction, SDG 13 (Climate Action) through the mitigation of greenhouse gas emissions associated with food waste, and SDG 9 (Industry, Innovation and Infrastructure) by encouraging the development of innovative technologies and sustainable industrial processes. Furthermore, the recovery of health-promoting bioactive compounds supports SDG 3 (Good Health and Well-being), while the adoption of circular bioeconomy strategies contributes to SDG 8 (Decent Work and Economic Growth) by fostering the development of new value chains and market opportunities.

Among the numerous compounds that can be recovered from agricultural residues, phytochemicals have attracted particular attention because of their diverse biological activities and potential health benefits [11,12]. Phytochemicals are naturally occurring secondary metabolites synthesized by plants, present also in agri-food waste, and include polyphenols, flavonoids, phenolic acids, anthocyanins, carotenoids, terpenoids, and other bioactive molecules [13]. These compounds are primarily involved in plant defense mechanisms and adaptation to environmental stress; however, they also exert beneficial effects in humans, including antioxidant, anti-inflammatory, antimicrobial, photoprotective, and anti-aging activities [14]. Remarkably, several studies have demonstrated that the concentration of these bioactive compounds is often higher in discarded plant fractions, such as peels, seeds, and pomaces, than in the edible portions commonly consumed [15,16,17].

This evidence has significantly increased scientific interest in the recovery and utilization of phytochemicals from agri-food by-products.

Among these bioactive constituents, antioxidant compounds are among the most valuable classes of molecules. Oxidative stress is widely recognized as a major contributor to cellular aging and the development of numerous chronic diseases. In the skin, excessive ROS production induced by ultraviolet radiation, environmental pollutants, and other external stressors accelerates the degradation of extracellular matrix components, promotes inflammation, impairs barrier function, and contributes to photoaging, pigmentation disorders, and loss of skin elasticity [18,19,20,21,22].

Consequently, antioxidants capable of neutralizing free radicals and modulating oxidative pathways have become essential ingredients in both preventive and therapeutic skin-care formulations [23,24,25,26].

Polyphenols and carotenoids are particularly relevant in this context due to their remarkable antioxidant capacity. Polyphenolic compounds, including flavonoids, tannins, and stilbenes, act as effective free-radical scavengers and metal chelators while also modulating intracellular signaling pathways involved in inflammation and cellular protection [27,28,29]. Similarly, carotenoids such as β-carotene, lycopene, lutein, and zeaxanthin provide protection against oxidative damage and ultraviolet-induced skin injury, contributing to the maintenance of skin homeostasis and overall skin health [30,31,32,33]. The multifunctional nature of these compounds makes them highly attractive ingredients for the formulation of innovative cosmeceutical products.

The cosmeceutical industry has experienced substantial growth over the past decade, driven by increasing consumer demand for natural, sustainable, and science-based products capable of improving skin appearance and health. Cosmeceuticals occupy the interface between cosmetics and pharmaceuticals, containing biologically active ingredients that provide benefits beyond basic skin care [34,35]. In response to growing concerns regarding the safety, sustainability, and environmental impact of synthetic ingredients, both consumers and manufacturers are increasingly seeking natural alternatives derived from renewable resources. Agri-food by-products represent an ideal source of such ingredients, offering a combination of sustainability, availability, cost-effectiveness, and high biological activity.

The successful exploitation of these resources has been facilitated by significant advances in extraction and processing technologies. Conventional extraction methods are progressively being complemented or replaced by environmentally friendly approaches designed to maximize extraction efficiency while minimizing solvent consumption, energy requirements, and environmental impact. Green extraction technologies, including ultrasound-assisted extraction (UAE), microwave-assisted extraction (MAE), supercritical fluid extraction (SFE), pressurized liquid extraction (PLE), and the use of natural deep eutectic solvents (NADES), have emerged as promising tools for the sustainable recovery of high-value bioactive compounds from agri-food residues [36,37,38,39,40]. These technologies are consistent with the principles of green chemistry and represent an essential component of modern waste valorization strategies.

In this context, the transformation of agricultural and food-processing by-products into antioxidant-rich ingredients for cosmeceutical applications represents a compelling example of how sustainability and innovation can converge to address environmental and societal challenges. By integrating waste valorization, green extraction technologies, and the development of natural bioactive ingredients, the agri-food and cosmetic sectors can contribute to a more circular, sustainable economy while simultaneously responding to consumer demand for effective, environmentally responsible products.

Although several reviews have addressed agri-food by-products, specific classes of bioactive compounds, or selected cosmeceutical applications, a comprehensive review integrating their potential use in skin, oral, and hair care is still lacking. Therefore, this review aims to provide a comprehensive overview of agri-food by-products as promising sources of antioxidant compounds for cosmeceutical applications. Particular attention will be devoted to the main classes of bioactive molecules recovered from these residues, innovative extraction technologies employed for their isolation, their mechanisms of action against oxidative stress, and their current and potential applications in cosmetic and skin-health formulations within the framework of sustainable development and circular bioeconomy principles. The available evidence is discussed from a mechanistic perspective, highlighting the biological pathways underlying the antioxidant effects of agri-food-derived bioactive compounds across skin, oral, and hair care applications.

## 2. Literature Search Strategy

A comprehensive literature search was conducted to identify studies investigating the recovery of antioxidant compounds from agri-food by-products and their potential applications in cosmeceutical formulations.

The search was performed in PubMed, Scopus, and Web of Science Core Collection databases and included articles published between January 2015 and June 2026. Earlier studies were considered when they represented seminal contributions or provided fundamental insights into the chemical characterization, biological activities, or mechanisms of action of the bioactive compounds investigated.

The search strategy combined controlled vocabulary and free-text terms using Boolean operators (AND/OR). The following keywords were used in different combinations: TITLE-ABS-KEY (“agri-food by-products” OR “food by-products” OR “food waste” OR “agricultural waste” OR “agro-industrial waste”) AND (antioxidant* OR polyphenol* OR flavonoid*) AND (cosmeceutical* OR cosmetic* OR skincare OR “skin care” OR “oral care” OR “hair care”)

Only peer-reviewed articles published in English were considered eligible. Original research articles, clinical studies, systematic reviews, and meta-analyses were included, whereas conference abstracts, editorials, letters, patents, and non-English publications were excluded. Although these eligibility criteria were established to ensure the quality and relevance of the included literature, the findings should be interpreted considering some methodological limitations. The literature search was restricted to three major bibliographic databases (PubMed, Scopus, and Web of Science Core Collection) and to English-language articles, which may have resulted in the omission of potentially relevant studies indexed elsewhere or published in other languages. In line with its narrative design, this review was intended to provide a critical and integrated overview of the available evidence rather than a systematic review.

Titles, abstracts, and full texts were independently screened by two authors to assess eligibility according to the predefined inclusion criteria. Any discrepancies were resolved through discussion and consensus.

**Figure 1:**
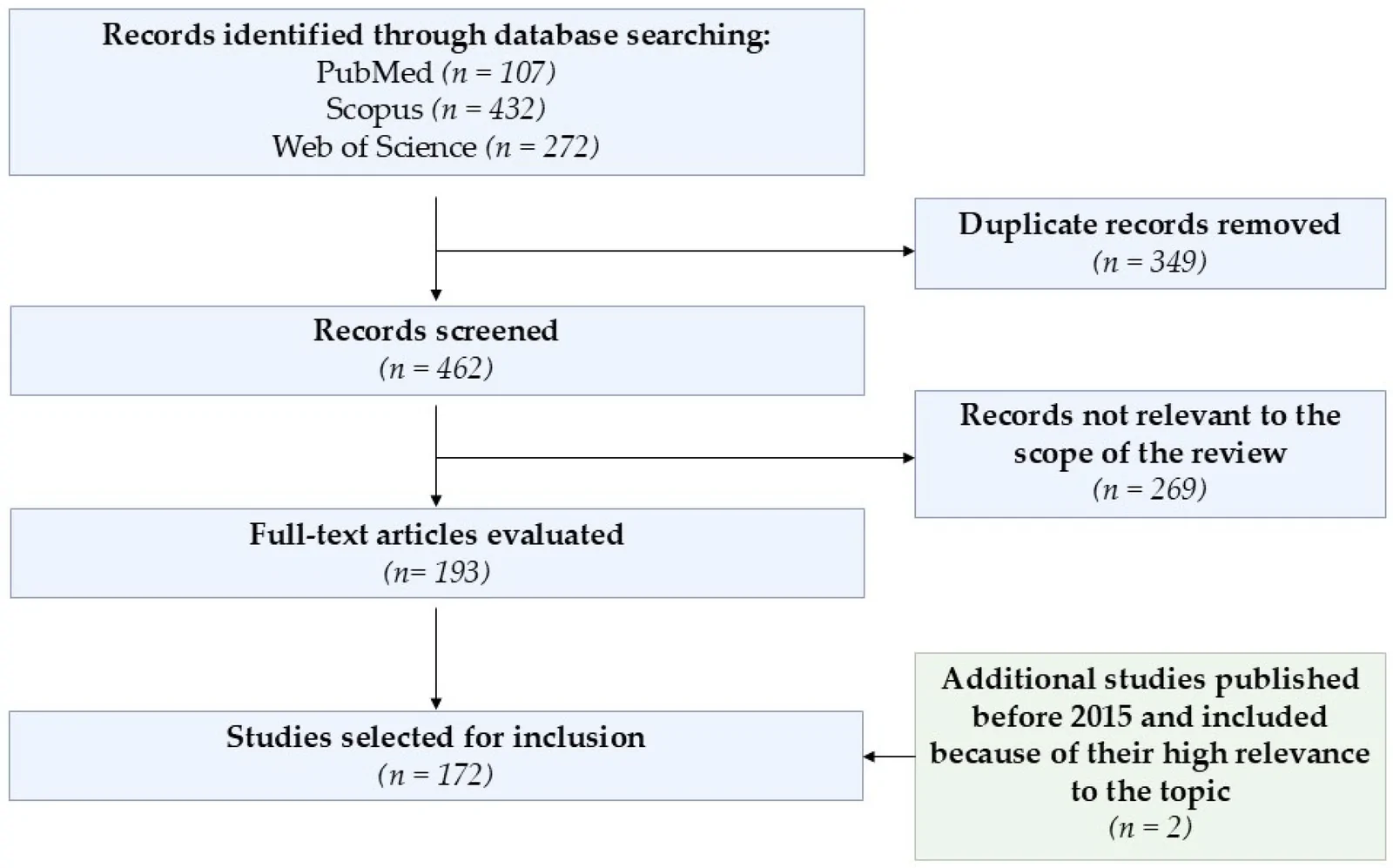
Overview of the literature search and study selection process.

An overview of the literature search and study selection process is presented in Figure 1.

## 3. Antioxidant Compounds in Agri-Food By-Products: Sources and Green Recovery Strategies

Agri-food by-products are a rich and diverse reservoir of antioxidant compounds, providing a sustainable and economically valuable source of bioactive molecules that can be recovered and reused in high-value applications [36,41,42].

The most abundant antioxidant compounds identified in agri-food by-products are phenolic compounds, which can be divided into flavonoids and non-flavonoids, the former including flavonols, flavones, flavanones, flavan-3-ols, anthocyanins, and isoflavones, and the latter mainly phenolic acids, stilbenes, lignans and tannins [37,43], as well as carotenoids (including lycopene, β-carotene and other provitamin A carotenoids) [44], and vitamins such as vitamin C (ascorbic acid), and vitamin E (tocopherols and tocotrienols) [45].

The distribution of antioxidant compounds across plant tissues is highly heterogeneous and depends on the botanical species, cultivar, environmental conditions, and processing methods [41]. Fruit and vegetable peels frequently contain significantly higher concentrations of phenolic compounds than the edible pulp, as these tissues constitute the first protective barrier against environmental stressors such as ultraviolet radiation, pathogens, and mechanical damage [43]. Citrus peels, for example, are particularly rich in phenolic acids, including caffeic, *p*-coumaric, ferulic, and sinapic acids, as well as flavonoids such as hesperidin and naringin [46,47]. Similarly, pomegranate peels contain high concentrations of hydrolyzable tannins, especially punicalagins and ellagitannins, together with gallic acid derivatives [48,49], while tomato skins represent an important source of carotenoids, particularly lycopene and β-carotene [48,50]. Seeds also represent a valuable source of antioxidant compounds [51]. Grape seeds are characterized by high levels of flavan-3-ols, catechins, epicatechins, and proanthocyanidins, which substantially contribute to their antioxidant potential [52,53]. Pomegranate seeds contain phenolic compounds, flavonoids, and lipid-soluble antioxidants, including tocopherols, phytosterols, and carotenoids [49,51].

Pomace, the solid residue remaining after juice, wine, or oil production, constitutes another valuable source of antioxidant compounds [54,55]. Grape pomace, one of the most extensively studied agro-industrial by-products, retains a significant proportion of the phenolic compounds originally present in grapes, since these compounds are not fully extracted during winemaking. These compounds include anthocyanins, flavanols, and resveratrol [55]. Apple pomace is particularly rich in chlorogenic acid, quercetin glycosides, phlorizin, and dietary fiber-associated antioxidants [56], whereas citrus pomace contains considerable amounts of flavonoids, phenolic acids, and residual vitamin C [54].

Olive oil pomace and related olive-processing by-products, including olive mill wastewater, olive leaves, and olive stone residues, are also particularly relevant sources of antioxidant phenolics, especially hydroxytyrosol, tyrosol, oleuropein derivatives, verbascoside, and secoiridoid compounds, which contribute to their strong radical-scavenging and anti-inflammatory potential [57,58]. The recovery of these compounds from olive oil by-products is of particular interest because olive processing generates large amounts of residues with high organic load, and their valorisation may reduce environmental impact while providing bioactive ingredients for functional foods, nutraceuticals, cosmetics, and active packaging applications [59].

Fibers recovered from agricultural pomace and other agro-industrial by-products represent another sustainable functional ingredient for antioxidant-rich food formulations and nutraceutical applications [60,61]. These matrices, including fruit and vegetable pomace, cereal bran, oilseed residues, and winery or olive-processing by-products, are often enriched in insoluble and soluble dietary fibers associated with bioactive compounds that may contribute to gut health, metabolic regulation, and modulation of oxidative stress [62,63]. Their valorization also supports circular-economy strategies by reducing agro-industrial waste while generating added-value health-oriented ingredients [64]. The remarkable diversity and abundance of antioxidant compounds in these residues highlight their potential as sustainable sources of high-value ingredients.

The efficient recovery of antioxidant compounds from agri-food by-products largely depends on the extraction strategy employed [36]. Because bioactive molecules are embedded within complex plant matrices and exhibit diverse physicochemical properties, including polarity, solubility, and stability, extraction conditions strongly influence both the yield and composition of the resulting extracts [65]. Conventional extraction techniques, typically using organic solvents such as methanol, ethanol, or acetone, or their aqueous mixtures, have long been used for their effectiveness in solubilizing phenolic compounds and other bioactive molecules [55]. However, these approaches are often associated with high solvent consumption, long extraction times, elevated energy requirements, and potential environmental and safety concerns. In recent years, increasing attention has been directed toward the development of sustainable and efficient extraction technologies that improve extraction yields while reducing environmental impact [41,66,67]. The most promising innovative green extraction techniques include: Supercritical Fluid Extraction (SFE), Microwave-Assisted Extraction (MAE), Ultrasound-Assisted Extraction (UAE), High-Pressure Homogenization (HPH), Pulsed Electric Fields (PEF), High Voltage Electrical Discharges (HVED), and the use of Natural Deep Eutectic Solvents (NaDES) [41,68,69]. These approaches enhance the release of intracellular bioactive compounds through different mechanisms, including cavitation, electromagnetic heating, pressure-induced disruption, electroporation, or solvent-mediated interactions, thereby improving mass transfer and extraction efficiency. In addition, many of these technologies reduce solvent consumption, shorten processing times, and help preserve thermolabile antioxidant compounds [69]. The main advantages and limits of these technologies are summarized in Table 1.

Consequently, selecting an appropriate extraction strategy for the recovery of antioxidants from agri-food waste will provide an environmentally friendly approach to obtain bioactive ingredients suitable for incorporation into innovative cosmetic and cosmeceutical products, while promoting resource efficiency and waste valorization.

## 4. Analytical Determination of Antioxidant Compounds Recovered from Agri-Food By-Products: The Contribution of Omics Technologies

The valorization of agri-food by-products for cosmeceutical applications requires both efficient recovery of bioactive compounds and reliable chemical characterization of the resulting extracts. These matrices are chemically complex and may vary according to plant species and cultivar, geographical origin, agronomic conditions, ripening stage, processing, storage, and extraction parameters. Consequently, conventional measurements of total phenolic content and antioxidant capacity, including Folin–Ciocalteu, DPPH, ABTS, FRAP, and ORAC assays, are useful for preliminary screening but are insufficient to establish extract identity, batch-to-batch reproducibility, or the compounds responsible for the observed biological effects.

Chromatographic and spectrometric techniques are therefore essential for compound-specific analysis. HPLC and UHPLC coupled with diode-array, fluorescence, or mass spectrometric detection are widely used to characterize phenolic acids, flavonoids, anthocyanins, stilbenes, secoiridoids, carotenoids, and other antioxidant constituents. Targeted LC–MS/MS methods allow sensitive quantification of selected markers using specific standards, whereas high-resolution mass spectrometry, including TOF- and Orbitrap-based platforms, supports broader structural annotation through accurate mass, isotopic patterns, and fragmentation spectra [93]. These approaches have been applied to the characterization of hydroxytyrosol, oleuropein derivatives, and other phenolics from olive oil-processing by-products [58], as well as to the quantification of flavanol glycosides in grape seeds and skins [53]. GC–MS is particularly suitable for volatile, semi-volatile, or derivatized compounds, while NMR spectroscopy provides complementary structural information and highly reproducible chemical fingerprints with limited sample preparation [94].

Within this framework, metabolomics is the omics approach most directly relevant to antioxidant-rich extracts. More broadly, foodomics integrates MS- and NMR-based platforms with different omics disciplines to investigate food composition, quality, traceability, functionality, and processing-related changes [95]. Targeted metabolomics quantifies predefined compounds, whereas untargeted metabolomics provides broader chemical fingerprints and enables the identification of discriminating metabolites. Chemometric tools, including principal component analysis and partial least-squares methods, can distinguish extracts, identify sources of variability, and select candidate quality-control markers. LC–HRMS- and NMR-based metabolomics therefore provide complementary approaches for the characterization and sustainable valorization of complex agri-food matrices [94,96].

Other omics approaches may also contribute. Proteomics and peptidomics can identify antioxidant peptides in protein-rich residues such as cereal bran, oilseed cakes, and dairy by-products, whereas lipidomics can characterize tocopherols, carotenoids, sterols, phospholipids, and unsaturated fatty acids. Transcriptomic and proteomic analyses may further reveal redox-sensitive, inflammatory, and stress-response pathways modulated by chemically characterized extracts.

However, untargeted omics data require cautious interpretation because compound annotation is often putative, matrix effects may alter signal intensity, and differences in analytical workflows may limit comparability. For cosmeceutical development, a tiered strategy combining preliminary antioxidant assays, untargeted fingerprinting, targeted quantification, contaminant and stability assessment, and correlation with biologically relevant endpoints is therefore preferable. This integrated approach is essential to define extract specifications, ensure reproducibility and safety, and support the development of standardized antioxidant ingredients from agri-food by-products.

## 5. Mechanisms of Action of Antioxidants from Agri-Food By-Products in Cosmeceutical Applications

Antioxidant components from agri-food by-products may contribute to counteract oxidative stress at the skin and oral mucosa levels. Excessive ROS/RNS levels not only damage lipids, proteins and DNA, but also act as second messengers that activate inflammatory transcription factors, mitochondrial stress responses, cellular senescence and extracellular matrix remodeling [22,97,98]. In oral mucosa, redox–inflammatory loops contribute to periodontal and gingival tissue damage, where microbial lipopolysaccharide triggers cytokine release and ROS generation [99].

Agri-food by-product extracts provide chemical antioxidants, including phenolics and carotenoids, capable of directly interacting with reactive species. Second, they may act as biological response modifiers by regulating endogenous antioxidant enzymes, redox-sensitive transcription factors and cytokine-driven pathways. Examples include lycopene-rich tomato skin, ellagitannin-rich pomegranate peel, flavanone-rich citrus peel, quercetin-rich onion peel and stilbene/flavanol-rich grape pomace [48,90,100,101,102,103].

As direct antioxidants, polyphenols can donate electrons or hydrogen atoms, chelate transition metals and interrupt radical chain reactions, whereas carotenoids quench singlet oxygen and protect lipid compartments from peroxidative damage [22,98,104]. This activity is especially relevant in the epidermis and stratum corneum, where oxidants generated in response to UV radiation and environmental pollutants target membrane lipids, proteins and matrix components [22]. In human primary gingival epithelial cells exposed to LPS, tomato skin and pomegranate peel extracts significantly reduced intracellular H_2_O_2_ and increased superoxide dismutase 1 (SOD1) expression [48]. Similarly, orange and lemon by-products extracts rich in hesperidin and related flavonoids reduced intracellular H_2_O_2_ in keratinocytes after oxidative challenge [90].

Independent evidence supports the broader relevance of citrus peel-derived bioactives. In UVB-irradiated HaCaT keratinocytes, an ethanol extract from immature *Citrus unshiu* peel reduced ROS accumulation, cellular senescence and apoptosis, and counteracted UVB-induced alterations in MMP-1, MMP-2, and MMP-9 expression [105].

Redox-sensitive inflammatory signalling represents a second major mechanism. Oxidative stress activates NF-κB, AP-1 and MAPK-related cascades, increasing cytokines, chemokines, inducible enzymes and adhesion molecules, including TNF-α, IL-1β, IL-6, IL-8, MCP-1, COX-2, iNOS and ICAM-1 [22,97,106,107]. This creates a self-amplifying loop in which inflammation promotes additional ROS production and oxidative damage. Independent evidence from oral models supports this redox–inflammatory framework. Hesperidin reduced *P. gingivalis*-induced ROS production in oral epithelial cells and attenuated NF-κB activation and the secretion of IL-1β, TNF-α, IL-8, MMP-2, and MMP-9 in *P. gingivalis*-stimulated macrophages [108]. A relevant by-product example is onion peel, a quercetin-rich residue: in LPS-stimulated RAW264.7 macrophages, the 50% ethanol extract of Allium cepa peel reduced nitric oxide (NO) and inducible nitric oxide synthase (iNOS), lowered IL-1α, IL-1β, IL-6 and IL-27, and directly inhibited the JAK/STAT pathway [101]. This is relevant for skin and mucosal applications because macrophages and keratinocytes share pattern-recognition and cytokine-driven pathways [97,109]. The broader quercetin literature further supports reductions in ROS, malondialdehyde (MDA), lipid peroxidation and inflammatory mediators, together with increases in glutathione, catalase and SOD [106].

Extracellular matrix preservation is a downstream consequence of redox–inflammatory control. UV-induced ROS and cytokine signaling up-regulate matrix metalloproteinases (MMPs), particularly MMP-1 and MMP-9, which degrade collagen and elastin and contribute to wrinkle formation, loss of firmness and dermal thinning [22,98,100,104]. Here, carotenoids and polyphenols are relevant as upstream modulators of ROS, AP-1/NF-κB and MMP expression, while the application-level evidence for lycopene-rich tomato interventions is discussed in the following section.

Carotenoid biology provides a useful mechanistic reference for lipid-phase photoprotection. A recent systematic review linked carotenoids to ROS/RNS scavenging, attenuation of chronic inflammation, modulation of MMPs, support of collagen biosynthesis, hydration and elasticity, and possible regulation of aquaporins and hyaluronic acid metabolism [98]. Astaxanthin and fucoxanthin have been demonstrated to influence mitochondrial resilience, NF-κB/NLRP3 activation, cytokine release, MMP expression, and senescence-associated markers [110,111].

Wound repair modulation should be interpreted in this same mechanistic frame. Early repair requires transient inflammatory signals for immune coordination, keratinocyte migration and re-epithelialization [97,112]. Accordingly, a previous study from our group showed that citrus waste-derived extracts that reduced H_2_O_2_ in HaCaT keratinocytes also enhanced wound closure and increased IL-6 and IL-8 release, a pattern compatible with early pro-regenerative signalling rather than a purely anti-inflammatory effect [90]. Resveratrol, available from grape skin and seeds and potentially recoverable from grape pomace, has also been associated with wound-healing, anti-scarring and anti-photoaging effects through antioxidant, anti-inflammatory and cell-protective mechanisms [112]. Indeed, grape pomace extract contributed to epidermal restoration, accelerated dermal turnover, and increased collagen synthesis in keratinocytes and fibroblasts [113].

UVA exposure reduces fibroblast viability, increases ROS, inflammatory mediators and MMPs, damages extracellular matrix and impairs autophagy. In human skin fibroblasts and mouse skin, resveratrol promoted AMP-activated protein kinase (AMPK) phosphorylation, activated autophagy, reduced ROS production, inhibited apoptosis, restored cell-cycle progression and attenuated UVA-induced photoaging [114]. These data are not specific to grape pomace extracts, but they support AMPK/autophagy as a candidate pathway for stilbene-rich by-product ingredients.

Pigmentation and barrier-related effects are best introduced as downstream consequences of redox and inflammatory regulation. ROS and RNS generated by UV can act as signalling mediators of melanogenesis, while inflammation contributes to post-inflammatory hyperpigmentation [22,106]. Hesperidin from orange peel is relevant because citrus by-products are abundant and hesperidin has been associated with antioxidant, anti-inflammatory, photoprotective, antibacterial and potentially anti-aging applications [100]. However, specific anti-pigmentation or barrier claims require melanocyte models, reconstructed epidermis, ex vivo skin or human data, rather than inference from antioxidant assays alone.

**Figure 2:**
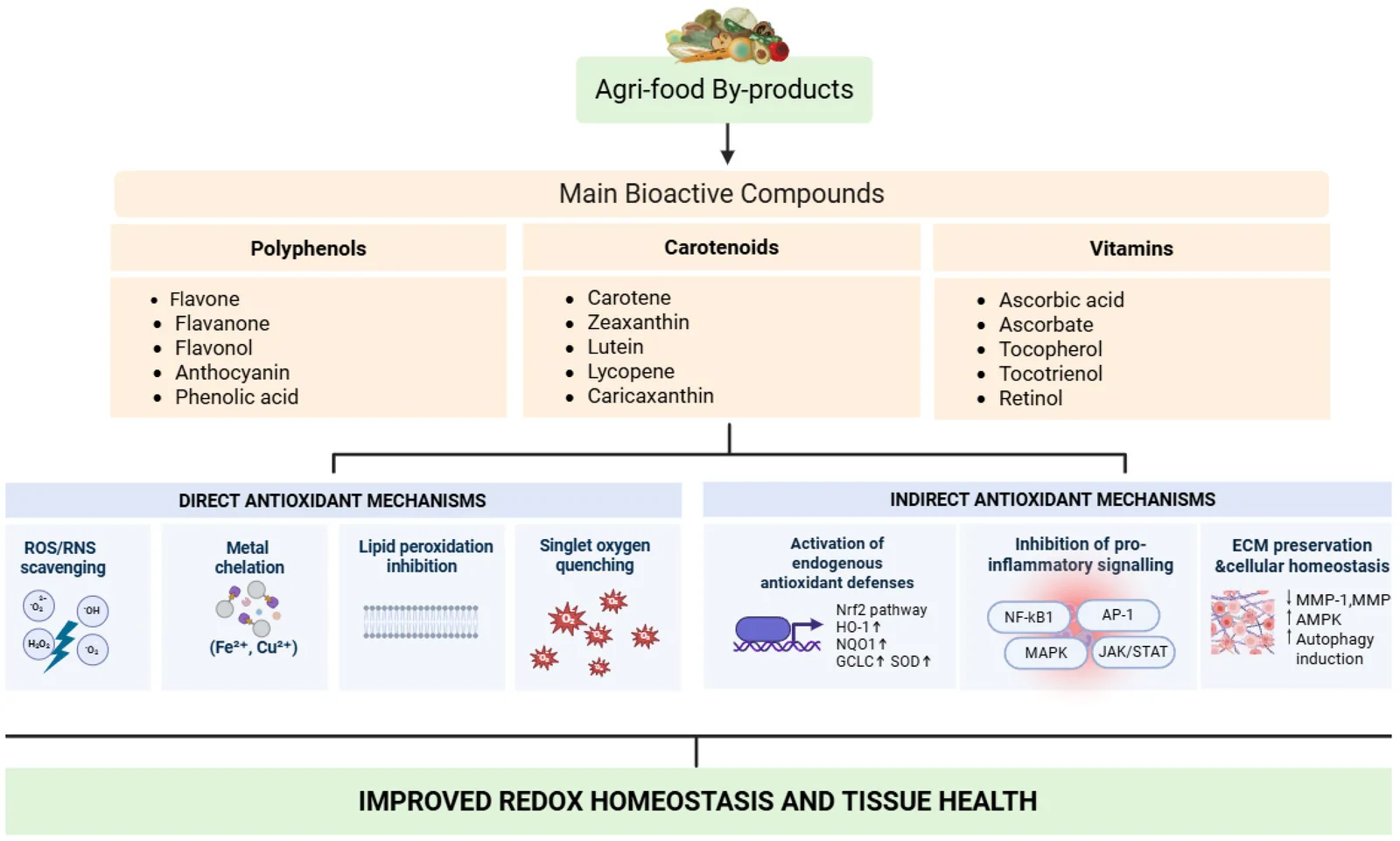
Schematic overview of the main bioactive compounds recovered from agri-food by-products and the principal mechanisms relevant to cosmeceutical applications, including direct ROS/RNS scavenging, modulation of endogenous antioxidant defenses and redox-sensitive signaling pathways, attenuation of inflammatory responses, preservation of extracellular matrix integrity, support of wound repair, and regulation of pigmentation. The figure summarizes the mechanisms discussed and referenced in Section 5. The figure is original and was created with BioRender.com (accessed on 30 June 2026).

Overall, agri-food by-product antioxidants in cosmeceutical applications should be conceptualized as multitarget bioactive agents. Their mechanisms include direct ROS/RNS scavenging, reduction in lipid peroxidation, support of endogenous antioxidant enzymes, possible Nrf2-mediated cytoprotection, suppression of NF-κB/AP-1/MAPK/JAK-STAT inflammatory signaling, reduction in cytokines and chemokines, attenuation of MMP-driven extracellular matrix degradation, protection from DNA damage, support of keratinocyte migration and wound healing, and possible regulation of pigmentation (Figure 2).

These mechanistic findings provide the rationale for the formulation- and application-oriented evidence discussed in the next sections. They should nevertheless be interpreted cautiously, because most data derive from in vitro or animal models and often use isolated compounds rather than standardized extracts [97].

## 6. Skin Care Applications

While the previous section focused on mechanisms, this section considers application-level evidence for anti-aging, hydration and anti-hyperpigmentation formulations. The most informative studies are those that evaluate defined extracts or finished formulations using cell models, reconstructed skin, ex vivo permeation, animal models or human instrumental endpoints. Across the available evidence, most extensively investigated agri-food by-products include citrus peels, apple pomace, grape pomace, olive pomace and grape seeds, tomato skin, pomegranate peel, onion peel, chestnut shell, kiwi peel, pineapple leaf fibre residues, and olive leaf (Table 2) [49,100,115,116,117,118,119,120,121,122]. Their cosmetic relevance extends beyond radical scavenging to humectant effects, matrix preservation, barrier support, pigment modulation, and protection against UV- and pollution-related stressors.

Apple pomace provides a useful example because progressed beyond chemical characterization toward formulation-level testing. Szymczak et al. evaluated an oil-in-water emulsion containing *Malus domestica* apple pomace extract and Roman chamomile hydrolate, combining polyphenol-rich antioxidant activity with soothing properties [115]. In a 14-day split-face study, the formulation improved skin hydration compared with baseline, supporting the use of pomace-derived polyphenols and polysaccharides in moisturizing anti-aging formulations [115]. Consistent in vitro data show that apple pomace extract increased human skin fibroblast proliferation and upregulated genes involved in hyaluronan synthesis, suggesting potential benefits for hydration and dermal matrix maintenance [123].

A second relevant formulation strategy involves the use of upcycled biopolymers, rather than relying exclusively on low-molecular-weight antioxidants. Shill et al. developed a Pineapple Leaf Fibre Crosspolymer (PALF) from discarded pineapple leaf fibres [116]. This ingredient is not primarily a classical antioxidant extract; rather, it combines film-forming, anti-pollution, hydration, and pigment-dispersion functions. In vitro and ex vivo studies showed that PALF reduced oxidative stress, attenuated inflammatory responses, protected against pollution and UV-related effects, and improved pigment dispersion. In vivo, PALF reduced dermal carbon deposition and improved skin hydration and barrier function [116]. This evidence expands the concept of antioxidant cosmeceuticals: some by-products may be valuable not only for delivering antioxidant molecules, but also for generating biopolymeric films that reduce contact with pollutants, improve water retention, and enhance formulation performance.

Citrus by-products are particularly relevant for anti-aging and anti-hyperpigmentation formulations because orange and lemon peels are abundant sources of flavanones. Hesperidin from orange peel has been proposed as a multifunctional skincare bioactive for anti-aging, barrier support, UV-induced damage, hyperpigmentation, and wound healing [100]. Independent experimental evidence further supports the photoprotective potential of citrus peel extracts. In UVB-irradiated HaCaT keratinocytes, an ethanol extract from immature *Citrus unshiu* peel attenuated ROS accumulation, cellular senescence, and apoptosis and counteracted UVB-induced alterations in MMP-1, MMP-2, and MMP-9 expression [105]. From a formulation perspective Stanisic et al., showed that hesperidin nanocrystals isolated from citrus peel can be incorporated into creams with skin-compatible properties, indicating that delivery technology is essential to overcome hesperidin’s low aqueous solubility and limited bioavailability [124]. For anti-hyperpigmentation, *Citrus unshiu* (also called Satsuma mandarin) peel extract has shown tyrosinase-inhibitory and melanin-reducing effects in vitro and skin-brightening effects in vivo models [125]. Nevertheless, claims of “skin whitening” or depigmentation should be carefully phrased and supported by controlled human studies using melanin index, colorimetry and standardized UV challenge models.

Pomegranate peel provides another strong example of a phenolic-rich by-product with anti-aging and anti-hyperpigmentation potential. Pomegranate peels are rich in ellagitannins, punicalagin, ellagic acid, gallic acid, and flavonoids, and recent reviews emphasize their antioxidant, anti-inflammatory, antimicrobial, photoprotective, and nanoparticle-forming potential [49]. It has been suggested that punicalagin has low skin permeability, making it more suitable for topical surface activity, whereas ellagic acid shows higher skin permeability and may be more relevant for sunscreen or anti-pigmentation formulations [49]. In vitro evidence indicates that punicalagin and ellagic acid derivatives can reduce melanogenesis through tyrosinase-related pathways, while pomegranate formulations have been reported to reduce UVB-induced DNA damage and support collagen-related endpoints in reconstituted skin [49]. Although human data remains limited, available findings are encouraging. Oral pomegranate juice or extract increased resistance to UVB-induced erythema and modified the skin microbiome in a randomized study of healthy women [126].

Grape-derived by-products are particularly relevant for anti-aging formulations thanks to their procyanidins, catechins, anthocyanins, and resveratrol content. In a previous study conducted by members of the present author team showed that grape pomace polyphenols extracted with NaDES could be incorporated into topical formulations, permeate skin models, and exert antioxidant and anti-inflammatory activity in 3D human keratinocytes [92]. This study is particularly important because the extraction solvent and delivery carrier are treated as part of a single formulation strategy. Clinical evidence is also emerging for grape seed extract. A sunscreen formulation reduced melanin and erythema and improved skin tone, hydration, and elasticity in skin [127]. A later clinical trial using grape seed extract-loaded hyalurosomes reported improved hydration, reduced wrinkle depth, and slight decreases in melanin and erythema levels, highlighting the value of nanocarrier-based delivery for polyphenol-rich by-products [128].

Olive oil pomace, olive leaves and olive mill wastewater are rich in phenolic compounds such as hydroxytyrosol, tyrosol, oleuropein derivatives and secoiridoids, and have been investigated as sustainable sources of antioxidant, anti-inflammatory, photoprotective and antimicrobial ingredients for topical skin-care and cosmeceutical formulations [121,122].

Tomato skin and tomato-processing residues are mainly relevant because of lycopene, a lipophilic carotenoid with strong singlet oxygen-quenching activity. Notably, tomato- and lycopene-based interventions are supported by strong human evidence than many other bioactive compounds derived from agri-food by-products. A systematic review and meta-analysis reported that tomato and lycopene interventions reduced UV-induced erythema-related parameters, MMP-1, ICAM-1, and skin pigmentation, and increased minimal erythema dose, skin thickness, and skin density [104]. These results support the use of lycopene-rich ingredients in anti-photoaging strategies, particularly in nutricosmetic or combined oral–topical approaches. However, the evidence is still more robust for dietary lycopene than for topical tomato skin extracts. For topical formulations, the main challenges are carotenoid oxidation, poor water compatibility, color impact, and delivery into lipid skin compartments.

Taken together, these studies indicate that formulation context determines whether antioxidant by-products can support anti-aging, hydration or pigmentation effects. Phenolic compounds are already used in skincare but are still at modest levels: a survey of 1299 products found phenolics in 13.2% of anti-aging products, 5.2% of sunscreens and 4.8% of aftersun products, despite strong mechanistic support for antioxidant, anti-inflammatory and anti-hyperpigmentation activity [129]. This gap likely reflects extract variability, limited skin penetration, instability, odor/color issues, regulatory uncertainty and insufficient clinical substantiation. Therefore, the strongest claims should be reserved for standardized ingredients tested in finished products with objective endpoints, including corneometry, transepidermal water loss, cutometry, profilometry, melanin and erythema indices, colorimetry, MMP/collagen biomarkers and standardized UV or pollution challenge models. Table 2 summarizes the biological activities of the main bioactive compounds derived from agri-food by-products in skin care applications.

## 7. Oral Care Applications

The oral cavity is a dynamic interface where interactions among the resident microbiota, host immune responses, and environmental factors are essential for maintaining tissue homeostasis. Disruption of this balance leads to oxidative stress, a key contributor to oral tissue dysfunction and disease progression.

Under physiological conditions, enzymatic and non-enzymatic antioxidant systems preserve redox balance within the oral environment and counteract ROS accumulation. However, chronic exposure to microbial dysbiosis, tobacco smoke, poor oral hygiene, aging, and systemic comorbidities can overwhelm these defense mechanisms, resulting in a sustained oxidative state [99]. Excessive ROS production contributes to the pathogenesis of a broad spectrum of oral disorders, including dental caries, periodontal diseases, oral mucositis, peri-implantitis, and oral potentially malignant disorders [99], by amplifying inflammatory responses, promoting extracellular matrix degradation, and compromising tissue homeostasis [130]. Consequently, maintaining oral redox homeostasis has emerged as an attractive target for preventive and adjunctive oral care strategies.

In this context, agri-food by-products represent sustainable and cost-effective sources of multifunctional bioactive compounds that can simultaneously target multiple pathogenic processes implicated in oral diseases. The main agri-food by-products explored as sources of antioxidant ingredients for oral cosmeceutical applications, along with their bioactive compounds, experimental oral models, and reported biological activities, are summarized in Table 3.

Among the agri-food by-products investigated for oral care applications, grape pomace has received considerable attention owing to its high content of proanthocyanidins, flavan-3-ols, anthocyanins, and stilbenes. Experimental studies have demonstrated that grape-derived proanthocyanidins exert antioxidant and anti-inflammatory effects [131], while inhibiting the growth, adhesion, and biofilm formation of major oral pathogens, including *Streptococcus mutans*, *Porphyromonas gingivalis*, *Fusobacterium nucleatum*, and *Aggregatibacter actinomycetemcomitans* [131,132]. These activities contribute to the preservation of extracellular matrix integrity, gingival tissue homeostasis, and oral microbial balance. In addition to proanthocyanidins, grape pomace contains stilbenes, particularly resveratrol, which has been shown to attenuate oxidative stress and reduce the expression of pro-inflammatory mediators in lipopolysaccharide-stimulated human gingival fibroblasts, thereby counteracting the onset of periodontal diseases [133]. Furthermore, oleanolic acid recovered from wine pomace has been shown to selectively reduce the salivary abundance of periodontopathic bacteria without significantly altering the overall composition of the oral microbiota, highlighting its potential as a microbiome-modulating ingredient [134].

Similar multifunctional properties have been reported for pomegranate peel, a rich source of ellagitannins, including punicalagin, punicalin, ellagic acid derivatives, and gallic acid. Pomegranate peel extracts exhibit broad-spectrum antimicrobial activity against cariogenic and periodontal pathogens, including *Lactobacillus acidophilus*, *Streptococcus mitis*, *Streptococcus mutans*, *Streptococcus salivarius*, *Streptococcus sanguinis*, and *Streptococcus oralis*, and *Candida albicans* [48,135]. Beyond their antimicrobial effects, a previous study conducted by members of the present author team showed that tomato skin and pomegranate peel extracts exerted antioxidant activity in LPS-challenged human gingival epithelial cells [48]. Independent studies further support related effects in oral models: pomegranate peel extract modulated the expression of multiple inflammatory mediators in gingiva-derived mesenchymal stromal cells [136], while hesperidin reduced *P. gingivalis*-induced ROS production and NF-κB activation in oral epithelial and macrophage models [108].

Olive oil processing residues, particularly olive pomace and olive mill wastewater, are rich sources of hydroxytyrosol, tyrosol, oleuropein, and related secoiridoids. These compounds exhibit potent antioxidant and anti-inflammatory properties and have demonstrated promising effects in preventing oral tissue damage. Hydroxytyrosol has been associated with reduced alveolar bone resorption and improved periodontal outcomes in in vivo models [137]. Tyrosol has recently shown antimicrobial activity against oral pathogens such as *Streptococcus mutans* and *Candida tropicalis*, further supporting the valorization of olive-derived by-products for oral applications [138,139].

Additional agri-food by-products with promising oral care applications include citrus processing residues, tea-processing waste, cocoa husks, and tomato pomace.

Citrus residues are abundant sources of flavanones such as hesperidin, naringenin, and narirutin, which have demonstrated anti-inflammatory and anti-biofilm activities in experimental models of oral disease [108,140]. In addition, these compounds impair *Streptococcus mutans* biofilm formation and reduce bacterial acidogenicity, suggesting a potential role in caries prevention [141].

Tea-processing waste and cocoa husks provide valuable sources of catechins, especially epigallocatechin-3-gallate, which exerts antioxidant, anti-inflammatory, and antimicrobial activities while improving salivary antioxidant capacity [142,143,144]. Similarly, tomato processing by-products, including peels and seeds, are important sources of lycopene, a carotenoid with exceptional singlet-oxygen-quenching capacity. Growing evidence indicates that lycopene may play a role in managing oral potentially malignant disorders (OPMDs), such as oral leukoplakia and oral submucous fibrosis. Clinical and preclinical studies have reported improvements in lesion size, hyperkeratosis, and antioxidant status following lycopene supplementation, while experimental models indicate that lycopene may interfere with pathways involved in apoptosis, cell proliferation, and malignant transformation [145].

Despite growing evidence supporting the biological activities of agri-food-derived bioactives, their translation into effective oral cosmeceutical products remains in its early stages. To date, only a limited number of studies have investigated the incorporation of waste-derived extracts into oral formulations. For instance, tomato skin and pomegranate peel extracts have recently been incorporated into a prototype mouthwash formulation that retained antioxidant, anti-inflammatory, and antibacterial activities in primary human gingival epithelial cells, supporting the feasibility of waste-derived oral care products [48]. Similarly, grape pomace-derived ingredients have been explored for use in freeze-dried mouthwashes and remineralizing formulations, showing promising physicochemical properties and biological activity in preclinical settings [146].

The translation of agri-food-derived bioactives into oral cosmeceuticals remains challenging owing to the unique characteristics of the oral environment, including salivary clearance, enzymatic degradation and the dynamic nature of oral biofilms, which collectively limit compound bioavailability and residence time. Therefore, advanced delivery systems, including nanoencapsulation and mucoadhesive platforms, together with standardized extraction procedures and rigorous quality-control strategies, will be essential to ensure compound stability, mucosal retention and reproducible biological activity.

**Table 3:** Agri-food by-products investigated as sources of bioactive compounds for oral care applications.

Agri-Food By-Product	Main Bioactive Compounds	Oral Models	Biological Activities	Key References
Grape pomace and grape seeds	Proanthocyanidins, flavan-3-ols, anthocyanins, resveratrol, oleanolic acid	*S. mutans*, *Porphyromonas gingivalis*, human gingival fibroblasts	ROS scavenging; inhibition of glucosyltransferases and biofilm formation; downregulation of IL-1β, IL-6, IL-8, TNF-α;	[131,132,133,134]
Pomegranate peel	Punicalagins, punicalin, ellagic acid, gallic acid, anthocyanins	*S. mutans*, *S. sanguinis*, *S. oralis*, *Candida albicans*, human gingival epithelial cells	Antioxidant activity; inhibition of NF-κB signaling; reduction in ROS production; antimicrobial and antibiofilm effects	[48,135,136]
Olive pomace and olive mill wastewater	Hydroxytyrosol, tyrosol, oleuropein, secoiridoids	Experimental periodontitis models; *S. mutans*; *Candida tropicalis*	Reduction in alveolar bone resorption; antimicrobial activity	[137,138,139]
Citrus processing residues	Hesperidin, naringenin, narirutin	*S. mutans* biofilms; inflammatory oral cell models	Anti-inflammatory activity; inhibition of bacterial adhesion and acidogenicity; suppression of biofilm maturation	[108,140,141]
Tea-processing waste	Catechins, epigallocatechin-3-gallate (EGCG)	Oral biofilm models; salivary antioxidant models	ROS scavenging; anti-inflammatory activity; inhibition of bacterial growth and biofilm formation; enhancement of salivary antioxidant capacity	[142,143]
Cocoa husks	Catechins, epicatechins, procyanidins, theobromine	Oral biofilm models, *S. mutans*	Antioxidant and antibiofilm effects; inhibition of bacterial adhesion	[143,144]
Tomato pomace (peels and seeds)	Lycopene, phenolic acids, flavonoids	Oral potentially malignant disorder (OPMD) models; human gingival epithelial cells	Singlet oxygen quenching; modulation of apoptosis and cell proliferation; reduction in oxidative stress and inflammatory signaling	[48,145]

## 8. Hair Care Applications

Hair has a fundamental role in physical appearance and psychosocial well-being, representing an important indicator of age, health, and social identity. Beyond its aesthetic function, the hair follicle (HF) is a dynamic, tiny organ that undergoes continuous cycles of growth (anagen), regression (catagen), and rest (telogen) [147]. In addition to hair production, HFs serve as a reservoir of stem cells that support skin homeostasis, wound healing, and tissue regeneration [148].

HF function is tightly regulated by complex interactions between epithelial and dermal compartments and is particularly sensitive to oxidative stress.

Indeed, oxidative stress is increasingly recognized as a key contributor to HF aging and hair loss disorders, including androgenetic alopecia (AGA). Excessive ROS production contributes to the impairment of dermal papilla cell function, induces cellular senescence, promotes inflammation, and alters signaling pathways involved in HF growth and regeneration [149,150,151]. Consequently, antioxidant compounds have emerged as promising candidate ingredients for hair care formulations. Growing interest has recently been directed toward agri-food by-products as sustainable sources of antioxidant compounds for hair care applications [149,152,153]. Table 4 summarizes the main agri-food by-products explored for potential hair care cosmeceutical applications.

Among these, coffee processing residues have attracted significant attention due to their abundance, with coffee production generating more than 20 million tons of waste annually [154]. Among these, coffee pulp, one of the main by-products of the coffee industry, is particularly rich in phenolic compounds, flavonoids, and caffeine, well-known for their positive biological activities [155]. Indeed, recent studies have demonstrated that coffee pulp extracts possess strong antioxidant activity and promote the proliferation and migration of human HF dermal papilla cells (HFDPCs), which are essential for HF development and cycling [156]. These effects were associated with the activation of hair growth-related pathways, including Wnt/β-catenin and Sonic Hedgehog signaling, and with the inhibition of 5α-reductase isoforms involved in androgen-dependent hair loss. Moreover, VEGF-associated signaling stimulation suggests a potential role in enhancing follicular vascularization and regeneration.

Similarly, rice bran, a major by-product of rice milling, has emerged as a valuable source of bioactive phytochemicals, including γ-oryzanol, tocopherols, ferulic acid, chlorogenic acid, and other polyphenolic compounds [157]. Extracts derived from rice bran have been shown to stimulate HFDPC proliferation while exerting antioxidant and anti-inflammatory effects [158]. In addition, they modulate key molecular pathways implicated in hair growth by suppressing 5α-reductase expression and activating Wnt/β-catenin, Sonic Hedgehog, and VEGF signaling. These findings suggest that rice bran-derived products may promote hair growth through a multifactorial mechanism involving protection against oxidative damage, attenuation of inflammation, and stimulation of follicular regenerative processes.

By-products obtained from *Camellia* species have also demonstrated considerable potential for hair care applications. Extracts from discarded *Camellia japonica* fruit shells were shown to promote dermal papilla cell (DPC) proliferation and activate VEGF- and Wnt-dependent pathways associated with follicular growth [157,159]. Furthermore, the extracts exhibited anti-androgenic activity by inhibiting 5α-reductase and reducing Dkk-1 expression, associated with hair loss and follicular miniaturization, respectively. Their strong antioxidant properties reduced intracellular ROS levels and prevented cellular senescence, thereby preserving follicular function and regenerative capacity.

Likewise, *Camellia oleifera* seed cake, a by-product of camellia oil production, traditionally used in Asian hair care practices, has emerged as a promising source of multifunctional bioactive compounds. Experimental studies demonstrated that seed cake extracts stimulate DPC proliferation through the activation of potassium channels and growth signaling pathways, including MAPKs and protein kinase Akt [160]. The extracts also modulate the expression of several growth factors involved in HF development, such as VEGF, IGF-1, and HGF. In addition to their growth-promoting properties, *C. oleifera* extracts exhibit anti-inflammatory, anti-apoptotic, anti-androgenic, and anti-senescent activities, thereby counteracting multiple mechanisms associated with androgenetic alopecia. Their efficacy has also been confirmed in vivo, where treatment improved hair density, hair shaft thickness, and follicular development [161].

Overall, current evidence suggests that agri-food by-products represent a promising and sustainable source of bioactive ingredients for hair care cosmeceuticals. Despite originating from diverse agricultural matrices, these materials appear to exert convergent biological effects by mitigating oxidative stress and inflammation, modulating androgen signaling, enhancing follicular vascularization, and stimulating pathways involved in hair growth and regeneration. Such multifunctional properties support their potential incorporation into next-generation formulations aimed at preventing hair loss and promoting scalp and hair health. However, it should be noted that current evidence supporting the use of agri-food by-products in hair care remains largely based on in vitro experiments and a limited number of animal studies. Indeed, no clinical trials have yet evaluated the efficacy of these waste-derived ingredients for promoting hair growth or preventing hair loss. Therefore, the available data should be considered preliminary, and well-designed randomized controlled clinical studies are needed to validate their efficacy, safety, and long-term benefits in humans.

**Table 4:** Agri-food by-products investigated as sources of bioactive compounds for hair care applications.

Agri-Food By-Product	Main Bioactive Compounds	Hair Models	Biological Activities	Key References
Coffee pulp	Phenolic compounds, flavonoids, caffeine	Human hair follicle dermal papilla cells	Antioxidant activity; promotion of cell proliferation and migration; activation of Wnt/β-catenin and Sonic Hedgehog pathways; inhibition of 5α-reductase isoforms (SRD5A1-3); stimulation of VEGF signaling and follicular vascularization	[155,156]
Rice bran	γ-Oryzanol, tocopherols, ferulic acid, chlorogenic acid, polyphenols	Human hair follicle dermal papilla cells; DU-145 cells	Promotion of cell proliferation; antioxidant and anti-inflammatory effects; inhibition of SRD5A1-3 expression; activation of Wnt/β-catenin, Sonic Hedgehog, and VEGF pathways; stimulation of hair follicle regeneration	[157,158]
Camellia japonica fruit shell	Gallic acid and protocatechuic acid	Human hair follicle dermal papilla cells	Increased proliferation; activation of VEGF and Wnt/β-catenin signaling; inhibition of 5α-reductase activity; suppression of Dkk-1 expression; ROS scavenging; protection against oxidative stress-induced senescence; anti-androgenic activity	[159]
Camellia oleifera seed cake	Polyphenols, flavonoids, tannins, alkaloids, saponins	Dermal papilla cells; C57BL/6J mice	Promotion of cell proliferation; activation of potassium channels; stimulation of ERK/MAPK and PI3K/Akt pathways; upregulation of VEGF, IGF-1, and HGF; anti-inflammatory, anti-apoptotic, anti-androgenic and anti-senescent effects; improved hair density, hair shaft thickness, and follicular development in vivo	[160,161]

## 9. Future Perspectives, Limitations and Challenges

Agri-food by-products are increasingly recognized as sustainable and cost-effective sources of antioxidant compounds with significant potential for cosmeceutical applications. However, despite the expanding body of research demonstrating the efficacy of bioactive molecules derived from fruit peels, seeds, pomaces, and other agricultural residues, important limitations related to standardization, stability, safety, and regulatory compliance still restrict their widespread industrial adoption [162]. Addressing these issues will be essential to fully exploit the potential of agri-food waste for the valorization of skin, oral, and hair care products.

Several agri-food-derived ingredients have progressed toward industrial or advanced formulation use in skin and oral care. Coffee by-product oils, fibers, and polyphenol-rich extracts have been developed as antioxidant, moisturizing, emollient, exfoliating, and barrier-support ingredients, including coffee-silverskin extracts obtained by scalable supercritical CO_2_ extraction [163,164]. Grape seed and pomace extracts have also been incorporated into topical delivery systems and prototype facial masks. In oral care, pomegranate peel extract has been tested in experimental toothpastes and fluoride-containing mouthwashes [165], while tomato skin and pomegranate peel extracts were included in a prototype mouthwash retaining antioxidant, anti-inflammatory, and antibacterial activity [48]. Despite this translational progress, most applications remain pre-commercial or at an early industrial stage. Wider adoption will depend on raw-material standardization, batch reproducibility, sensory properties, stability, formulation compatibility, bioavailability, regulatory compliance, and controlled clinical validation of standardized finished products.

One of the main limitations to the widespread use of agri-food by-products is the substantial variability in their phytochemical profiles. The qualitative and quantitative composition of antioxidant bioactive molecules is strongly dependent on factors such as cultivar selection, geographical provenance, agricultural management, climatic conditions, harvesting time, and post-harvest handling. As a result, ensuring reproducible extraction performance and consistent product quality remains challenging, representing a critical barrier to industrial implementation and regulatory approval [166].

Another critical challenge is the stability of natural antioxidants during extraction, processing, storage, and incorporation into cosmetic formulations. Many bioactive compounds, including polyphenols, carotenoids, and vitamins, are highly sensitive to light, oxygen, temperature, and pH variations, which can lead to degradation and reduced biological activity. Moreover, the incorporation of antioxidant compounds into cosmeceutical formulations requires delivery approaches that can ensure their effective, controlled, and safe release at the target site within the skin [167]. At the same time, protecting these bioactive molecules from degradation during formulation, storage, and application is crucial for preserving their biological activity and maximizing their therapeutic potential. Future research should focus on the development of advanced stabilization strategies, such as nanoencapsulation, liposomal delivery systems, microencapsulation, and the use of biodegradable carriers capable of protecting bioactive molecules and enhancing their bioavailability and efficacy.

The economic feasibility of green extraction technologies also requires further investigation. Although environmentally friendly approaches such as ultrasound-assisted extraction, microwave-assisted extraction, supercritical fluid extraction, and deep eutectic solvents have shown promising results, their transition from laboratory to industrial scale remains challenging due to equipment costs and process optimization requirements. As summarized in Table 1, the scalability of green extraction technologies is an important aspect. Although SFE consistently produced high-purity extracts with limited solvent residues, its industrial implementation remains constrained by the high capital investment required for high-pressure equipment, elevated operational costs, and energy consumption. Likewise, high-voltage electrical discharge (HVED), despite its ability to efficiently disrupt plant tissues and enhance polyphenol recovery without excessive thermal degradation, still faces technological barriers related to reactor design, energy efficiency, and continuous-flow processing. These engineering challenges explain why both SFE and HVED are currently adopted primarily for high-value ingredients rather than for large-volume production of cosmetic raw materials. The critical challenges associated with process scale-up need a link between proof-of-concept studies and industrial applications [168]. The synthesis of current knowledge and future perspectives highlights the potential of green extraction technologies to enable the sustainable, cost-effective recovery of natural antioxidants from agri-food waste, supporting the transition toward a circular and resource-efficient bioeconomy.

Safety assessment represents another crucial aspect. While agri-food by-products are generally considered safe, the presence of contaminants, pesticide residues, mycotoxins, heavy metals, or allergenic compounds cannot be excluded [169,170]. Therefore, rigorous quality control procedures and toxicological evaluations should be performed before their incorporation into commercial cosmeceutical products. Long-term studies evaluating skin compatibility, sensitization potential, oral mucosal safety, and possible adverse reactions are still limited and warrant further investigation.

Regulatory issues constitute an additional barrier to market penetration. The regulatory framework governing cosmetic and cosmeceutical ingredients varies among countries and often lacks specific guidelines for bioactive compounds recovered from agri-food waste [171,172]. Harmonized regulations and clear quality specifications will be necessary to facilitate the approval and commercialization of upcycled ingredients. Furthermore, greater transparency regarding ingredient sourcing, traceability, and sustainability claims will be required to meet both regulatory requirements and consumer expectations.

A key limitation is also the relatively small number of well-designed clinical studies. Most of the available evidence is derived from in vitro experiments and animal models, whereas human trials remain scarce. Future research should focalize on randomized, controlled clinical studies aimed at validating the efficacy of agri-food-derived antioxidants in improving skin aging, pigmentation disorders, oral health conditions, scalp health, and hair growth.

Looking ahead, advances in omics technologies, artificial intelligence, and precision formulation approaches may accelerate the discovery of novel bioactive compounds and optimize their application in personalized cosmeceutical products. Integrating sustainable extraction methods with innovative delivery systems and robust clinical validation could significantly enhance the value of agri-food by-products, transforming waste into high-value ingredients. Ultimately, overcoming current limitations through multidisciplinary collaboration among researchers, industry stakeholders, and regulatory authorities will be essential to promote the widespread adoption of safe, effective, and sustainable antioxidant-based cosmeceuticals.

In addition, future studies should systematically incorporate life cycle assessment (LCA) and techno-economic assessment (TEA) to evaluate the overall sustainability of antioxidant recovery processes. While many green extraction technologies have demonstrated promising extraction efficiencies at laboratory scale, comprehensive evaluations of their environmental footprint, including energy consumption, water use, solvent recovery, greenhouse gas emissions, and waste generation, remain limited. Integrating environmental and economic indicators with extraction performance and biological efficacy will enable a more realistic comparison among technologies and support the selection of processes that are not only efficient but also environmentally and economically sustainable. Such multidisciplinary assessments will be essential for translating laboratory-scale innovations into commercially viable and circular bioeconomy-oriented cosmeceutical production.

## 10. Conclusions

The increasing focus on sustainability and the circular economy has identified agri-food by-products as valuable sources of bioactive compounds for cosmeceutical applications. Peels, seeds, pomaces, and other food-processing residues are rich in antioxidants, including polyphenols, flavonoids, carotenoids, and vitamins, which can counteract oxidative stress and promote skin, oral, and hair health. Advances in green and environmentally friendly extraction technologies have improved the recovery efficiency of these compounds while reducing environmental impact. Such approaches not only contribute to waste valorization, but also support the development of sustainable ingredients aligned with current consumer demand for natural and eco-friendly cosmetic products.

The biological activities of antioxidants derived from agri-food by-products are supported by a growing body of evidence demonstrating their ability to neutralize ROS, modulate inflammatory responses, and regulate cellular signaling pathways involved in tissue homeostasis and aging. These mechanisms are involved in the beneficial effects in various cosmeceutical applications, including anti-aging formulations, skin hydration, protection against hyperpigmentation, oral tissue protection, antimicrobial activity, and the prevention of oxidative damage affecting hair follicles and scalp health. Moreover, the multifunctional nature of many agri-food waste-derived antioxidants offers opportunities for the development of innovative formulations with combined antioxidant, anti-inflammatory, and antimicrobial properties.

Overall, the comparative assessment of the available evidence indicates that fruit peels and pomaces, particularly those derived from citrus, grape, pomegranate, tomato, and olive processing, are among the most extensively investigated sources of antioxidant compounds for cosmeceutical applications. Their prominence is largely associated with their high content of polyphenols, flavonoids, carotenoids, and secoiridoids, together with the availability of established recovery and characterization procedures. However, the level of evidence and the degree of clinical translation differ considerably among the different agri-food by-products. Most candidates are supported primarily by chemical characterization and in vitro studies, whereas only a limited number have progressed to in vivo investigation, clinical assessment, or evaluation in finished formulations. A formal quantitative synthesis across the different by-products was not feasible because of substantial heterogeneity in raw materials, extraction procedures, extract standardization, experimental models, concentrations, exposure conditions, and biological endpoints. Therefore, the potential of a given by-product should not be evaluated solely on the basis of its antioxidant capacity, but also by considering extract reproducibility, chemical standardization, formulation performance, safety, and the level of translational validation.

From a translational perspective, comprehensive toxicological assessments, regulatory harmonization, and long-term clinical studies are still required to ensure safety, reproducibility, and consumer confidence. Moreover, the strength of the supporting evidence also varies substantially across the different application fields. Skin care currently represents the most advanced area, with several ingredients already evaluated in human studies and formulation-based clinical trials. In contrast, evidence for oral care remains largely limited to in vitro investigations, with only a few clinical studies available, while hair care research is still almost exclusively based on preclinical models.

In conclusion, this narrative review provides an integrated mechanistic perspective on the potential of antioxidant compounds recovered from agri-food by-products for skin, oral, and hair care applications. By highlighting the shared biological mechanisms underlying these apparently distinct fields, it offers a comprehensive framework for understanding the cosmeceutical potential of agri-food-derived bioactive compounds and identifies common opportunities for future research and product development. Beyond their biological relevance, the valorization of agri-food by-products represents a promising strategy to simultaneously address environmental sustainability, support the circular economy, and foster innovation in the cosmetic and personal care sectors. Better interdisciplinary collaboration among researchers, industry stakeholders, and regulatory authorities will be essential to fully unlock the potential of these resources and facilitate their transition from laboratory research to commercial applications.

## Figures and Tables

**Table 1:** Main green extraction technologies for recovering antioxidant compounds from agri-food by-products: extraction mechanisms, target antioxidant classes, advantages, and limitations.

Extraction Method	Main Mechanism	Main Target Antioxidants	Advantages	Limitations	References
Ultrasound-Assisted Extraction (UAE)	Acoustic cavitation generates microbubbles that collapse near plant tissues, disrupting cell walls and enhancing mass transfer	Phenolic acids (e.g., gallic acid, caffeic acid), flavan-3-ols (e.g., epigallocatechin, catechin), anthocyanins, tannins, carotenoids	Low temperature, reduced extraction time, low equipment cost, efficient extraction of thermolabile compounds	Separation and purification steps required, non-uniform energy distribution, possible degradation under prolonged treatment, scale-up challenges	[70,71,72]
Microwave-Assisted Extraction (MAE)	Rapid dielectric heating of polar molecules generates internal pressure, leading to cell wall disruption and enhanced mass transfer	Flavonols (e.g., quercetin), anthocyanins, carotenoids, stilbenes (e.g., resveratrol)	High efficiency, short time, low solvent consumption; good reproducibility, easy scale-up	High equipment cost, possible thermal degradation of sensitive compounds, limited suitability for highly volatile molecules	[73,74,75,76]
Pulsed Electric Fields (PEF)	Short high-voltage pulses induce membrane electroporation, increasing cell permeability	Flavonoids (e.g., naringin), anthocyanins (e.g., malvidin-3-O-glucoside), tannins	Non-thermal process, high selectivity, low energy consumption, suitable as pre-treatment before extraction	High equipment cost, effectiveness depends on tissue conductivity and structure	[77,78,79,80]
High Voltage Electrical Discharges (HVED)	Electrical discharges generate shock waves, cavitation, turbulence, and electroporation, resulting in extensive cell disruption and enhanced mass transfer	Phenolic acids (e.g., caffeic acid), flavanones (e.g., naringin), anthocyanins (e.g., malvidin-3-O-glucoside), tannins	High extraction yields, low solvent consumption, efficient recovery of thermolabile compounds	Generation of reactive species may oxidize target compounds, hard to scale up, process optimization is required	[79,81,82]
High-Pressure Homogenization (HPH)	High shear forces, turbulence, and pressure gradients mechanically disrupt cellular structures	Phenolic acids (e.g., gallic acid, chlorogenic acid), flavonoids, carotenoids (e.g., lycopene, β-carotene)	High extraction yields, high scalability, effective disruption of rigid plant tissues, suitable for aqueous systems	High energy consumption, cooling needed, low extraction selectivity	[83,84]
Supercritical Fluid Extraction (SFE)	Solubilization of compounds in supercritical fluids (mainly CO_2_), with tunable solvent properties through pressure and temperature control, enabling selective extraction of target compounds	Carotenoids (e.g., lycopene, β-carotene), tocopherols, terpenes, lipophilic antioxidants, polyphenols (when co-solvents are used)	Solvent-free extracts, no toxic residues, high purity, excellent for thermolabile lipophilic compounds	High equipment costs, elevated pressure requirements, risk of volatile compounds losses	[85,86,87,88]
Natural Deep Eutectic Solvents (NaDES)	Hydrogen-bond-based solvent systems enhance the solubilization and stabilization of bioactive compounds, improving extraction efficiency	Phenolic acids, flavanones (e.g., hesperidin), anthocyanins (e.g., malvidin), tannins, stilbenes, carotenoids and tocopherols (with hydrophobic NaDES)	Biodegradable, low toxicity, tunable polarity, high extraction efficiency, enhanced stability of antioxidant compounds, compatible with UAE and MAE	High viscosity may limit mass transfer, difficult solvent recovery, limited industrial standardization, extraction efficiency depends on composition and water content	[72,89,90,91,92]

**Table 2:** Agri-food by-products investigated as sources of bioactive compounds for skin care applications.

Agri-Food By-Product	Main Bioactive Compounds	Skin Models	Biological Activities	Key References
Apple pomace	Polyphenols, polysaccharides	Human skin fibroblasts; human volunteers (14-day split-face study)	Antioxidant activity; increased fibroblast proliferation; upregulation of hyaluronan synthesis-related genes; improved skin hydration;	[115,123]
Pineapple leaf fiber residues	Cellulosic fibers, lignin-derived polyphenols	Human skin fibroblasts; human volunteers (14-day split-face study)	Anti-pollution effects; antioxidant and anti-inflammatory activity; improved hydration and barrier function; enhanced pigment dispersion; reduced dermal carbon deposition	[116]
Citrus peels	Hesperidin, naringin, flavanones, phenolic acids	Keratinocytes; Human skin fibroblasts; melan-a cells; guinea pigs	Anti-aging; photoprotection; barrier support; reduction in UV-induced oxidative damage, cellular senescence and apoptosis; modulation of MMP expression; tyrosinase inhibition; melanin reduction; skin-brightening effects	[100,105,124,125]
Pomegranate peel	Punicalagin, ellagic acid, ellagitannins, gallic acid, flavonoids	In vitro assays; reconstructed skin models; human volunteers (oral pomegranate juice for 12 weeks)	Antioxidant, anti-inflammatory, antimicrobial and photoprotective activities; inhibition of melanogenesis; reduction in UVB-induced DNA damage; support of collagen-related pathways	[48,49,126]
Grape pomace/grape seeds	Procyanidins, catechins, anthocyanins, resveratrol	3D human keratinocytes; skin permeation models; human volunteers (6-week or 12-week trials)	Antioxidant and anti-inflammatory activity; improved hydration and elasticity; reduced wrinkle depth, melanin and erythema;	[92,127,128]
Tomato skin and tomato-processing residues	Lycopene, carotenoids	Human intervention studies; systematic review and meta-analysis	Photoprotection; reduction in UV-induced erythema; decreased MMP-1 and ICAM-1; reduced pigmentation; increased minimal erythema dose, skin thickness and density	[104]
Onion peel	Quercetin, flavonoids, phenolic compounds	In vitro skin models; topical formulation studies	Antioxidant and anti-inflammatory effects;	[118]
Chestnut shell	Tannins, phenolic acids, flavonoids	In vitro skin models	Antioxidant and anti-inflammatory activities; protection against oxidative stress-related skin damage; potential anti-aging applications	[117]
Kiwi peel	Polyphenols, flavonoids, vitamin C-related compounds	In vitro assays	Antioxidant and skin-protective properties	[119]
Olive pomace, olive leaves and olive mill wastewater	Hydroxytyrosol, tyrosol, oleuropein, secoiridoids, flavonoids, phenolic acids	HaCaT keratinocytes; L929 fibroblasts; reconstructed human epidermis; cream formulations; human volunteers	Antioxidant and photoprotective activity; reduction in ROS formation in keratinocytes exposed to H_2_O_2_ or UVB; elastase inhibition; potential anti-aging and photoprotector-booster effects; good skin compatibility in topical formulations	[121,122]

## Data Availability

No new data were created or analyzed in this study. Data sharing is not applicable to this article.

## References

[1] Rao S., Nagendra A. (2026). An Insight into Food Waste Generations: Status, Source, and Global Impact. Microbial Cell Factories in Food Waste Biorefinery.

[2] Guo X., Broeze J., Groot J.J., Axmann H., Vollebregt M. (2020). A Worldwide Hotspot Analysis on Food Loss and Waste, Associated Greenhouse Gas Emissions, and Protein Losses. Sustainability.

[3] Adedeji A.A. (2022). Agri-Food Waste Reduction and Utilization: A Sustainability Perspective. J. ASABE.

[4] Priya K., Rani J., Gwal S. (2025). Transforming Agricultural Residues to Value-Added Products: Waste to Wealth. Sustainable Management of Agro-Food Waste.

[5] Hrelia S., Angeloni C., Barbalace M.C. (2023). Agri-Food Wastes as Natural Source of Bioactive Antioxidants. Antioxidants.

[6] Pal P., Singh A.K., Srivastava R.K., Rathore S.S., Sahoo U.K., Subudhi S., Sarangi P.K., Prus P. (2024). Circular Bioeconomy in Action: Transforming Food Wastes into Renewable Food Resources. Foods.

[7] Carus M., Dammer L. (2018). The Circular Bioeconomy—Concepts, Opportunities, and Limitations. Ind. Biotechnol..

[8] Tan E.C.D., Lamers P. (2021). Circular Bioeconomy Concepts—A Perspective. Front. Sustain..

[9] Food and Agricolture Organization of the United Nations (FAO) Sustainable Development Goals. https://www.fao.org/sustainable-development-goals/en/.

[10] Ferraz D., Pyka A. (2023). Circular Economy, Bioeconomy, and Sustainable Development Goals: A Systematic Literature Review. Environ. Sci. Pollut. Res..

[11] Fernandes F., Delerue-Matos C., Grosso C. (2025). Unveiling the Potential of Agrifood By-Products: A Comprehensive Review of Phytochemicals, Bioactivities and Industrial Applications. Waste Biomass Valorization.

[12] Dillard C.J., German J.B. (2000). Phytochemicals: Nutraceuticals and Human Health. J. Sci. Food Agric..

[13] Galali Y., Omar Z.A., Sajadi S.M. (2020). Biologically Active Components in By-products of Food Processing. Food Sci. Nutr..

[14] Sorrenti V., Burò I., Consoli V., Vanella L. (2023). Recent Advances in Health Benefits of Bioactive Compounds from Food Wastes and By-Products: Biochemical Aspects. Int. J. Mol. Sci..

[15] Parra-Pacheco B., Cruz-Moreno B.A., Aguirre-Becerra H., García-Trejo J.F., Feregrino-Pérez A.A. (2024). Bioactive Compounds from Organic Waste. Molecules.

[16] Castrica M., Rebucci R., Giromini C., Tretola M., Cattaneo D., Baldi A. (2019). Total Phenolic Content and Antioxidant Capacity of Agri-Food Waste and by-Products. Ital. J. Anim. Sci..

[17] Sagar N.A., Pareek S., Sharma S., Yahia E.M., Lobo M.G. (2018). Fruit and Vegetable Waste: Bioactive Compounds, Their Extraction, and Possible Utilization. Compr. Rev. Food Sci. Food Saf..

[18] Papaccio F., D′Arino A., Caputo S., Bellei B. (2022). Focus on the Contribution of Oxidative Stress in Skin Aging. Antioxidants.

[19] Rinnerthaler M., Bischof J., Streubel M., Trost A., Richter K. (2015). Oxidative Stress in Aging Human Skin. Biomolecules.

[20] Chen X., Yang C., Jiang G. (2021). Research Progress on Skin Photoaging and Oxidative Stress. Adv. Dermatol. Allergol..

[21] Salminen A., Kaarniranta K., Kauppinen A. (2022). Photoaging: UV Radiation-Induced Inflammation and Immunosuppression Accelerate the Aging Process in the Skin. Inflamm. Res..

[22] Chen J., Liu Y., Zhao Z., Qiu J. (2021). Oxidative Stress in the Skin: Impact and Related Protection. Int. J. Cosmet. Sci..

[23] Kumar S., Subba D.P., Seema, Firdous S.M., Odeku O.A., Kumar S., Sindhu R.K. (2025). Antioxidants in Skin Disorders. Antioxidants.

[24] Budzianowska A., Banaś K., Budzianowski J., Kikowska M. (2025). Antioxidants to Defend Healthy and Youthful Skin—Current Trends and Future Directions in Cosmetology. Appl. Sci..

[25] Kumar V., Tanwar N., Goel M., Khan M., Kumar D., Singh G., Mundlia J., Khatri N., Kumar A. (2025). Antioxidants for Skin Health. Recent Adv. Food Nutr. Agric..

[26] Azevedo Martins T.E., Sales de Oliveira Pinto C.A., Costa de Oliveira A., Robles Velasco M.V., Gorriti Guitiérrez A.R., Cosquillo Rafael M.F., Tarazona J.P.H., Retuerto-Figueroa M.G. (2020). Contribution of Topical Antioxidants to Maintain Healthy Skin—A Review. Sci. Pharm..

[27] Farjadmand F., Karimpour-Razkenari E., Nabavi S.M., Ardekani M.R.S., Saeedi M. (2021). Plant Polyphenols: Natural and Potent UV-Protective Agents for the Prevention and Treatment of Skin Disorders. Mini Rev. Med. Chem..

[28] Menaa F., Menaa A., Tréton J. (2014). Polyphenols against Skin Aging. Polyphenols in Human Health and Disease.

[29] Sun M., Deng Y., Cao X., Xiao L., Ding Q., Luo F., Huang P., Gao Y., Liu M., Zhao H. (2022). Effects of Natural Polyphenols on Skin and Hair Health: A Review. Molecules.

[30] Flieger J., Raszewska-Famielec M., Radzikowska-Büchner E., Flieger W. (2024). Skin Protection by Carotenoid Pigments. Int. J. Mol. Sci..

[31] Ma Y., Li C., Su W., Sun Z., Gao S., Xie W., Zhang B., Sui L. (2025). Carotenoids in Skin Photoaging: Unveiling Protective Effects, Molecular Insights, and Safety and Bioavailability Frontiers. Antioxidants.

[32] Varghese R., Emerson A., Vannier B., George Priya Doss C., Priyadharshini R., Efferth T., Ramamoorthy S. (2025). Substantial Effects of Carotenoids on Skin Health: A Mechanistic Perspective. Phytother. Res..

[33] Bin-Jumah M., Alwakeel S.S., Moga M., Buvnariu L., Bigiu N., Zia-Ul-Haq M. (2021). Application of Carotenoids in Cosmetics. Carotenoids: Structure and Function in the Human Body.

[34] Rathod S., Mali S., Shinde N., Aloorkar N. (2020). Cosmeceuticals and Beauty Care Products: Current Trends with Future Prospects. Res. J. Top. Cosmet. Sci..

[35] Crespi O., Rosset F., Pala V., Sarda C., Accorinti M., Quaglino P., Ribero S. (2025). Cosmeceuticals for Anti-Aging: Mechanisms, Clinical Evidence, and Regulatory Insights—A Comprehensive Review. Cosmetics.

[36] Yadav S., Malik K., Moore J.M., Kamboj B.R., Malik S., Malik V.K., Arya S., Singh K., Mahanta S., Bishnoi D.K. (2024). Valorisation of Agri-Food Waste for Bioactive Compounds: Recent Trends and Future Sustainable Challenges. Molecules.

[37] Panzella L., Moccia F., Nasti R., Marzorati S., Verotta L., Napolitano A. (2020). Bioactive Phenolic Compounds from Agri-Food Wastes: An Update on Green and Sustainable Extraction Methodologies. Front. Nutr..

[38] Vicente-Zurdo D., Gómez-Mejía E., Morante-Zarcero S., Rosales-Conrado N., Sierra I. (2025). Analytical Strategies for Green Extraction, Characterization, and Bioactive Evaluation of Polyphenols, Tocopherols, Carotenoids, and Fatty Acids in Agri-Food Bio-Residues. Molecules.

[39] Li J., Pettinato M., Campardelli R., De Marco I., Perego P. (2022). High-Pressure Technologies for the Recovery of Bioactive Molecules from Agro-Industrial Waste. Appl. Sci..

[40] Ristivojević P., Krstić Ristivojević M., Stanković D., Cvijetić I. (2024). Advances in Extracting Bioactive Compounds from Food and Agricultural Waste and By-Products Using Natural Deep Eutectic Solvents: A Circular Economy Perspective. Molecules.

[41] Ingallina C., Spano M., Prencipe S.A., Vinci G., Di Sotto A., Ambroselli D., Vergine V., Crestoni M.E., Di Meo C., Zoratto N. (2025). Enhancing Human Health Through Nutrient and Bioactive Compound Recovery from Agri-Food By-Products: A Decade of Progress. Nutrients.

[42] Vanacore D., Beltrami S., Gori A., Alderotti F., Lo Piccolo E., Detti C., Baptista A., Ferrini F., Brunetti C. (2025). Valorisation of Mediterranean Basin Fruit By-Products: Bioactive Composition, Applications, and Future Research Directions. Nat. Prod. Res..

[43] Ribas-Agustí A., Martín-Belloso O., Soliva-Fortuny R., Elez-Martínez P. (2018). Food Processing Strategies to Enhance Phenolic Compounds Bioaccessibility and Bioavailability in Plant-Based Foods. Crit. Rev. Food Sci. Nutr..

[44] Tomas M., Kamiloglu S., Nemli E., Ozdal T., Haque S., Apak R., Capanoglu E. (2025). From Carotene-Rich Waste-to-Food: Extraction, Food Applications, Challenges and Opportunities. Trends Food Sci. Technol..

[45] Timón M.L., Andrés A.I., Petrón M.J. (2024). Antioxidant Activity of Aqueous Extracts Obtained from By-Products of Grape, Olive, Tomato, Lemon, Red Pepper and Pomegranate. Foods.

[46] Singh B., Singh J.P., Kaur A., Singh N. (2020). Phenolic Composition, Antioxidant Potential and Health Benefits of Citrus Peel. Food Res. Int..

[47] Silla A., Punzo A., Caliceti C., Barbalace M.C., Hrelia S., Malaguti M. (2025). The Role of Antioxidant Compounds from Citrus Waste in Modulating Neuroinflammation: A Sustainable Solution. Antioxidants.

[48] Silla A., Punzo A., Bonvicini F., Perillo M., Malaguti M., Lorenzini A., Foltran I., Mercatante D., Mandrioli M., Rodriguez-Estrada M.T. (2025). Anti-Inflammatory, Antioxidant and Antibacterial Properties of Tomato Skin and Pomegranate Peel Extracts: A Sustainable Approach for Oral Health Care. Antioxidants.

[49] Debnath M., Aneja D. (2025). Waste Valorization Utilizing Green Nanotechnology: A Sustainable Approach for Pomegranate Peel Agro Wastes in Skincare Formulations. Bioresour. Bioprocess..

[50] Maciejczyk E., Wajs-Bonikowska A., Batory M., Budzisz E. (2025). Tomato Pomace: Underestimated Sustainable Cosmetic/Pharmaceutical Raw Source. Molecules.

[51] Fernandes D.S., Ferreira Neto G.M., Galiciani G.G., Ferreira R.d.S., Santana L.F., Hiane P.A., do Nascimento V.A., Pott A., Guimarães R.d.C.A., Freitas K.d.C. (2026). Fruit Seeds with Functional Applications: From Food Waste to Potential Uses. Molecules.

[52] Lucarini M., Durazzo A., Romani A., Campo M., Lombardi-Boccia G., Cecchini F. (2018). Bio-Based Compounds from Grape Seeds: A Biorefinery Approach. Molecules.

[53] Zerbib M., Cazals G., Enjalbal C., Saucier C. (2018). Identification and Quantification of Flavanol Glycosides in Vitis Vinifera Grape Seeds and Skins during Ripening. Molecules.

[54] Spedale C., Chiofalo V., Dell’Anno M., Frazzini S., Puglia M., Pedrazzi S., Moscatelli A., Onelli E., Rossi L. (2026). Sustainable Valorization of Citrus Pomace Waste: Biochar Production and in Vitro Bioactivities. Vet. Res. Commun..

[55] Wang J., Du P., Ju H., Li M., Cai Y., Yu Q., Liu X., Lv Y. (2026). Recent Advances in the Extraction and Skincare Applications of Grape By-Product Extracts. Nanoscale.

[56] Mensah R.Q., Gao Y., Dunn K., Strong J., Ball M., Plaza F. (2026). Biokinetic Profiling of Nutritional Value and Bioactivity in Grape and Apple Pomace during Microbial Proliferation. Bioresour. Technol..

[57] Mattioli L.B., Corazza I., Budriesi R., Hrelia S., Malaguti M., Caliceti C., Amoroso R., Maccallini C., Crupi P., Clodoveo M.L. (2024). From Waste to Health: Olive Mill Wastewater for Cardiovascular Disease Prevention. Nutrients.

[58] Abbattista R., Ventura G., Calvano C.D., Cataldi T.R.I., Losito I. (2021). Bioactive Compounds in Waste By-Products from Olive Oil Production: Applications and Structural Characterization by Mass Spectrometry Techniques. Foods.

[59] Teksoy A., Şen M. (2025). Evaluation of Olive Processing Wastes in Terms of Zero Discharge and Green Energy Production. Sci. Rep..

[60] Saikia S., Mahanta C.L. (2016). In Vitro Physicochemical, Phytochemical and Functional Properties of Fiber Rich Fractions Derived from by-Products of Six Fruits. J. Food Sci. Technol..

[61] Prata C., Zalambani C., Rossi F., Rossello S., Cerchiara T., Cappadone C., Malucelli E. (2025). Nutrients and Nutraceuticals from Vitis Vinifera L. Pomace: Biological Activities, Valorization, and Potential Applications. Nutrients.

[62] Fărcaș A.C., Socaci S.A., Nemeș S.A., Pop O.L., Coldea T.E., Fogarasi M., Biriș-Dorhoi E.S. (2022). An Update Regarding the Bioactive Compound of Cereal By-Products: Health Benefits and Potential Applications. Nutrients.

[63] Jagelavičiūtė J., Bašinskienė L., Čižeikienė D. (2025). Enhancing the Nutritional Value of Foods Through Probiotics and Dietary Fiber from Fruit and Berry Pomace. Fermentation.

[64] Gómez-García R., Campos D.A., Aguilar C.N., Madureira A.R., Pintado M. (2021). Valorisation of Food Agro-Industrial by-Products: From the Past to the Present and Perspectives. J. Environ. Manag..

[65] Boateng I.D. (2023). Recent Advances in Combined Avant-Garde Technologies (Thermal-Thermal, Non-Thermal-Non-Thermal, and Thermal-Non-Thermal Matrix) to Extract Polyphenols from Agro Byproducts. J. Food Drug Anal..

[66] Sun Q., Huang M., Zeng J., Ma M., Li J., Le T. (2025). Beyond Waste to Bioactive Potential: Advances in Olive by-Product Valorization and Resource Recovery. Food Chem..

[67] Vilas-Boas A.A., Magalhães D., Gómez-García R., Campos D.A., Correia M., Pintado M. (2025). Upcycled Orange Peel Ingredients: A Scoping Review on Phytochemical Composition, Extraction Techniques, and Biorefinery Strategies. Foods.

[68] Hndeya A.G., Sbhatu D.B., Gebreyohannes G. (2026). Advances in Eco-Friendly Extraction of Fruit Bioactive Compounds: Technologies, Challenges and Future Directions. Anal. Sci. Adv..

[69] Carpentieri S., Soltanipour F., Ferrari G., Pataro G., Donsì F. (2021). Emerging Green Techniques for the Extraction of Antioxidants from Agri-Food By-Products as Promising Ingredients for the Food Industry. Antioxidants.

[70] Ahmad T., Masoodi F.A., Rather S.A., Wani S.M., Gull A. (2019). Supercritical Fluid Extraction: A Review. J. Biol. Chem. Chron..

[71] Murador D.C., Braga A.R.C., Martins P.L.G., Mercadante A.Z., de Rosso V.V. (2019). Ionic Liquid Associated with Ultrasonic-Assisted Extraction: A New Approach to Obtain Carotenoids from Orange Peel. Food Res. Int..

[72] De los Ángeles Fernández M., Espino M., Gomez F.J.V., Silva M.F. (2018). Novel Approaches Mediated by Tailor-Made Green Solvents for the Extraction of Phenolic Compounds from Agro-Food Industrial by-Products. Food Chem..

[73] Vinatoru M., Mason T.J., Calinescu I. (2017). Ultrasonically Assisted Extraction (UAE) and Microwave Assisted Extraction (MAE) of Functional Compounds from Plant Materials. TrAC Trends Anal. Chem..

[74] Varadharajan V., Shanmugam S., Ramaswamy A. (2017). Model Generation and Process Optimization of Microwave-assisted Aqueous Extraction of Anthocyanins from Grape Juice Waste. J. Food Process Eng..

[75] Pal C.B.T., Jadeja G.C. (2019). Microwave-Assisted Deep Eutectic Solvent Extraction of Phenolic Antioxidants from Onion (*Allium cepa* L.) Peel: A Box–Behnken Design Approach for Optimization. J. Food Sci. Technol..

[76] Pinela J., Prieto M.A., Carvalho A.M., Barreiro M.F., Oliveira M.B.P.P., Barros L., Ferreira I.C.F.R. (2016). Microwave-Assisted Extraction of Phenolic Acids and Flavonoids and Production of Antioxidant Ingredients from Tomato: A Nutraceutical-Oriented Optimization Study. Sep. Purif. Technol..

[77] Niu D., Ren E.-F., Li J., Zeng X.-A., Li S.-L. (2021). Effects of Pulsed Electric Field-Assisted Treatment on the Extraction, Antioxidant Activity and Structure of Naringin. Sep. Purif. Technol..

[78] Niu D., Zeng X.-A., Ren E.-F., Xu F.-Y., Li J., Wang M.-S., Wang R. (2020). Review of the Application of Pulsed Electric Fields (PEF) Technology for Food Processing in China. Food Res. Int..

[79] Barba F.J., Brianceau S., Turk M., Boussetta N., Vorobiev E. (2015). Effect of Alternative Physical Treatments (Ultrasounds, Pulsed Electric Fields, and High-Voltage Electrical Discharges) on Selective Recovery of Bio-Compounds from Fermented Grape Pomace. Food Bioproc. Tech..

[80] Ahmad Shiekh K., Odunayo Olatunde O., Zhang B., Huda N., Benjakul S. (2021). Pulsed Electric Field Assisted Process for Extraction of Bioactive Compounds from Custard Apple (*Annona squamosa*) Leaves. Food Chem..

[81] Dalvi-Isfahan M., Hamdami N., Le-Bail A., Xanthakis E. (2016). The Principles of High Voltage Electric Field and Its Application in Food Processing: A Review. Food Res. Int..

[82] El Kantar S., Rajha H.N., Boussetta N., Vorobiev E., Maroun R.G., Louka N. (2019). Green Extraction of Polyphenols from Grapefruit Peels Using High Voltage Electrical Discharges, Deep Eutectic Solvents and Aqueous Glycerol. Food Chem..

[83] Jurić S., Ferrari G., Velikov K.P., Donsì F. (2019). High-Pressure Homogenization Treatment to Recover Bioactive Compounds from Tomato Peels. J. Food Eng..

[84] Zhu X., Cheng Y., Chen P., Peng P., Liu S., Li D., Ruan R. (2016). Effect of Alkaline and High-Pressure Homogenization on the Extraction of Phenolic Acids from Potato Peels. Innov. Food Sci. Emerg. Technol..

[85] Da Porto C., Natolino A. (2017). Supercritical Fluid Extraction of Polyphenols from Grape Seed (*Vitis vinifera*): Study on Process Variables and Kinetics. J. Supercrit. Fluids.

[86] Hatami T., Meireles M.A.A., Ciftci O.N. (2019). Supercritical Carbon Dioxide Extraction of Lycopene from Tomato Processing By-Products: Mathematical Modeling and Optimization. J. Food Eng..

[87] Pinto D., de la Luz Cadiz-Gurrea M., Sut S., Ferreira A.S., Leyva-Jimenez F.J., Dall’Acqua S., Segura-Carretero A., Delerue-Matos C., Rodrigues F. (2020). Valorisation of Underexploited Castanea Sativa Shells Bioactive Compounds Recovered by Supercritical Fluid Extraction with CO2: A Response Surface Methodology Approach. J. CO2 Util..

[88] Martinez G.A., Rebecchi S., Decorti D., Domingos J.M.B., Natolino A., Del Rio D., Bertin L., Da Porto C., Fava F. (2016). Towards Multi-Purpose Biorefinery Platforms for the Valorisation of Red Grape Pomace: Production of Polyphenols, Volatile Fatty Acids, Polyhydroxyalkanoates and Biogas. Green Chem..

[89] Kagueyam S.S., dos Santos Filho J.R., Contato A.G., de Souza C.G.M., Castoldi R., Corrêa R.C.G., Conte Junior C.A., Yamaguchi N.U., Bracht A., Peralta R.M. (2025). Green Extraction of Bioactive Compounds from Plant-Based Agri-Food Residues: Advances Toward Sustainable Valorization. Plants.

[90] Silla A., Punzo A., Comito R., Porru E., Gozzi G., Barbalace M.C., Perillo M., Lorenzini A., Malaguti M., Hrelia S. (2025). Upcycling of Citrus Waste by Natural Deep Eutectic Solvents: Green Extraction of Bioactive Compounds with Antioxidant and Regenerative Properties on Human Keratinocytes. Nutrients.

[91] Rashid R., Mohd Wani S., Manzoor S., Masoodi F.A., Masarat Dar M. (2023). Green Extraction of Bioactive Compounds from Apple Pomace by Ultrasound Assisted Natural Deep Eutectic Solvent Extraction: Optimisation, Comparison and Bioactivity. Food Chem..

[92] Punzo A., Porru E., Silla A., Simoni P., Galletti P., Roda A., Tagliavini E., Samorì C., Caliceti C. (2021). Grape Pomace for Topical Application: Green NaDES Sustainable Extraction, Skin Permeation Studies, Antioxidant and Anti-Inflammatory Activities Characterization in 3D Human Keratinocytes. Biomolecules.

[93] Roda A., Michelini E., Caliceti C., Guardigli M., Mirasoli M., Simoni P. (2018). Advanced Bioanalytics for Precision Medicine. Anal. Bioanal. Chem..

[94] Ciampa A., Danesi F., Picone G. (2022). NMR-Based Metabolomics for a More Holistic and Sustainable Research in Food Quality Assessment: A Narrative Review. Appl. Sci..

[95] Muguruma Y., Nunome M., Inoue K. (2022). A Review on the Foodomics Based on Liquid Chromatography Mass Spectrometry. Chem. Pharm. Bull..

[96] Donno D., Mellano M.G., Gamba G., Riondato I., Beccaro G.L. (2020). Analytical Strategies for Fingerprinting of Antioxidants, Nutritional Substances, and Bioactive Compounds in Foodstuffs Based on High Performance Liquid Chromatography–Mass Spectrometry: An Overview. Foods.

[97] Fernandes A., Rodrigues P.M., Pintado M., Tavaria F.K. (2023). A Systematic Review of Natural Products for Skin Applications: Targeting Inflammation, Wound Healing, and Photo-Aging. Phytomedicine.

[98] Stanescu C., Chiscop I., Mihalache D., Popa F., Tamas C., Stoleriu G. (2025). Skin Aging and Carotenoids: A Systematic Review of Their Multifaceted Protective Mechanisms. Nutrients.

[99] Kumar J., Teoh S.L., Das S., Mahakknaukrauh P. (2017). Oxidative Stress in Oral Diseases: Understanding Its Relation with Other Systemic Diseases. Front. Physiol..

[100] Rodrigues C.V., Pintado M. (2024). Hesperidin from Orange Peel as a Promising Skincare Bioactive: An Overview. Int. J. Mol. Sci..

[101] Lee H.-S., Kwon Y.-J., Seo E.-B., Kim S.-K., Lee H., Lee J.-T., Chang P.-S., Choi Y.J., Lee S.-H., Ye S.-K. (2023). Anti-Inflammatory Effects of *Allium cepa* L. Peel Extracts via Inhibition of JAK-STAT Pathway in LPS-Stimulated RAW264.7 Cells. J. Ethnopharmacol..

[102] Durante M., Montefusco A., Marrese P.P., Soccio M., Pastore D., Piro G., Mita G., Lenucci M.S. (2017). Seeds of Pomegranate, Tomato and Grapes: An Underestimated Source of Natural Bioactive Molecules and Antioxidants from Agri-Food by-Products. J. Food Compos. Anal..

[103] Singh B., Singh J.P., Kaur A., Singh N. (2018). Phenolic Compounds as Beneficial Phytochemicals in Pomegranate (*Punica granatum* L.) Peel: A Review. Food Chem..

[104] Zhang X., Zhou Q., Qi Y., Chen X., Deng J., Zhang Y., Li R., Fan J. (2024). The Effect of Tomato and Lycopene on Clinical Characteristics and Molecular Markers of UV-Induced Skin Deterioration: A Systematic Review and Meta-Analysis of Intervention Trials. Crit. Rev. Food Sci. Nutr..

[105] Piao M.J., Kang K.A., Fernando P.D.S.M., Herath H.M.U.L., Senavirathna H.M.M.M., Kang H.K., Hyun J.W. (2025). Anti-Senescence and Anti-Apoptotic Effects of Immature Citrus Unshiu Peel Ethanol Extract on Ultraviolet B-Irradiated Skin Keratinocytes. Toxicol. Res..

[106] Okselni T., Septama A.W., Juliadmi D., Dewi R.T., Angelina M., Yuliani T., Saragih G.S., Saputri A. (2025). Quercetin as a Therapeutic Agent for Skin Problems: A Systematic Review and Meta-Analysis on Antioxidant Effects, Oxidative Stress, Inflammation, Wound Healing, Hyperpigmentation, Aging, and Skin Cancer. Naunyn. Schmiedebergs. Arch. Pharmacol..

[107] Bang E., Kim D.H., Chung H.Y. (2021). Protease-Activated Receptor 2 Induces ROS-Mediated Inflammation through Akt-Mediated NF-ΚB and FoxO6 Modulation during Skin Photoaging. Redox Biol..

[108] Maquera-Huacho P.M., Spolidorio D.P., Manthey J., Grenier D. (2023). Effect of Hesperidin on Barrier Function and Reactive Oxygen Species Production in an Oral Epithelial Cell Model, and on Secretion of Macrophage-Derived Inflammatory Mediators during Porphyromonas Gingivalis Infection. Int. J. Mol. Sci..

[109] Jiang Y., Tsoi L.C., Billi A.C., Ward N.L., Harms P.W., Zeng C., Maverakis E., Kahlenberg J.M., Gudjonsson J.E. (2020). Cytokinocytes: The Diverse Contribution of Keratinocytes to Immune Responses in Skin. JCI Insight.

[110] Zhou X., Cao Q., Orfila C., Zhao J., Zhang L. (2021). Systematic Review and Meta-Analysis on the Effects of Astaxanthin on Human Skin Ageing. Nutrients.

[111] Fernando P.D.S.M., Kang K.A., Piao M.J., Herath H.M.U.L., Senavirathna H.M.M.M., Kim E.T., Kim H.-S., Chae S., Park M., Hyun J.W. (2025). Fucoxanthin Ameliorates PM _2.5_ -Mediated Skin Cell Inflammation and Senescence. Toxicol. Mech. Methods.

[112] Hecker A., Schellnegger M., Hofmann E., Luze H., Nischwitz S.P., Kamolz L., Kotzbeck P. (2022). The Impact of Resveratrol on Skin Wound Healing, Scarring, and Aging. Int. Wound J..

[113] Crăciun L.M., Dumitriu B.G., Olariu L., Jurcoane S., Cristea S., Adil A., Rosoiu N., Papacocea R. (2019). Regenerative and Scare Healing Potential of Active Compounds from Camelina Sativa Oil and Grape Pomace. Rom. Biotechnol. Lett..

[114] Xia Y., Zhang H., Wu X., Xu Y., Tan Q. (2024). Resveratrol Activates Autophagy and Protects from UVA-Induced Photoaging in Human Skin Fibroblasts and the Skin of Male Mice by Regulating the AMPK Pathway. Biogerontology.

[115] Szymczak K., Krajewska A., Grzyb M., Jodłowska I., Mietlińska K., Bonikowski R. (2026). Upcycling Roman Chamomile Hydrolate and Apple Pomace Agri-Wastes into Sustainable Cosmetic Ingredients. Antioxidants.

[116] Shill D.D., Goodson K.C., Stade H.N., Sneed C.M., Haller D., Drohan M., Drumwright M.D., Scholz D. (2026). From Crown to Cosmetics: Exploring the Efficacy of an Upcycled Pineapple Biopolymer in Sustainable Skincare, Suncare and Hair Care Applications. Int. J. Cosmet. Sci..

[117] Gomes S.M., Campos F., Martins M.C.L., Monteiro C., Santos L. (2025). Exploring the Bioactive Potential and Biocompatibility of Extracts from Agro-Industrial Residues for Cosmetic Applications. Int. J. Mol. Sci..

[118] Martinez S.F., Jaouhari Y., Giovannelli L., Bordiga M. (2026). From Waste to Dermocosmetic Value: A Narrative Review of Agro-Industrial Residues in Skincare Innovation. Appl. Sci..

[119] Silletta A., Mancuso A., d’Avanzo N., Cristiano M.C., Paolino D. (2024). Antimicrobial Compounds from Food Waste in Cosmetics. Cosmetics.

[120] Krzyżostan M., Wawrzyńczak A., Nowak I. (2024). Use of Waste from the Food Industry and Applications of the Fermentation Process to Create Sustainable Cosmetic Products: A Review. Sustainability.

[121] Nunes A., Marto J., Gonçalves L., Martins A.M., Fraga C., Ribeiro H.M. (2022). Potential Therapeutic of Olive Oil Industry By-products in Skin Health: A Review. Int. J. Food Sci. Technol..

[122] Nunes A., Gonçalves L., Marto J., Martins A.M., Silva A.N., Pinto P., Martins M., Fraga C., Ribeiro H.M. (2021). Investigations of Olive Oil Industry By-Products Extracts with Potential Skin Benefits in Topical Formulations. Pharmaceutics.

[123] Nanashima N., Maeda H., Nakajima A., Nishizuka M., Narumi T., Ichita J., Itoku K. (2024). Apple Pomace Extract Induces Cell Proliferation and Increases Type I Collagen and Hyaluronan Production in Human Skin Fibroblasts In Vitro. Plant Foods Hum. Nutr..

[124] Stanisic D., Liu L.H.B., dos Santos R.V., Costa A.F., Durán N., Tasic L. (2020). New Sustainable Process for Hesperidin Isolation and Anti-Ageing Effects of Hesperidin Nanocrystals. Molecules.

[125] Kim J.-K., Park N.-H., Hwang J.-S. (2019). Skin Lightening Effect of the Dietary Intake of Citrus Peel Extract Against UV-Induced Pigmentation. Nat. Prod. Commun..

[126] Henning S.M., Yang J., Lee R.-P., Huang J., Hsu M., Thames G., Gilbuena I., Long J., Xu Y., Park E.H. (2019). Pomegranate Juice and Extract Consumption Increases the Resistance to UVB-Induced Erythema and Changes the Skin Microbiome in Healthy Women: A Randomized Controlled Trial. Sci. Rep..

[127] Yarovaya L., Waranuch N., Wisuitiprot W., Khunkitti W. (2022). Clinical Study of Asian Skin Changes after Application of a Sunscreen Formulation Containing Grape Seed Extract. J. Cosmet. Dermatol..

[128] Rached R.A., Habre M., Salem Y., Khodeir J., Allaw M., Castangia I., Rajha H.N., Habre L., Feghali J., Touma J.A. (2025). Clinical Trial to Evaluate the Effect of Grape Seed Extract-Loaded Hyalurosomes on Skin Wellness. Cosmetics.

[129] Jesus A., Ratanji S., Cidade H., Sousa E., Cruz M.T., Oliveira R., Almeida I.F. (2025). Phenolics as Active Ingredients in Skincare Products: A Myth or Reality?. Molecules.

[130] Jain A., Bahuguna R. (2015). Role of Matrix Metalloproteinases in Dental Caries, Pulp and Periapical Inflammation: An Overview. J. Oral Biol. Craniofac. Res..

[131] Chen H., Wang W., Yu S., Wang H., Tian Z., Zhu S. (2022). Procyanidins and Their Therapeutic Potential against Oral Diseases. Molecules.

[132] Esteban-Fernández A., Zorraquín-Peña I., Ferrer M.D., Mira A., Bartolomé B., González de Llano D., Moreno-Arribas M.V. (2018). Inhibition of Oral Pathogens Adhesion to Human Gingival Fibroblasts by Wine Polyphenols Alone and in Combination with an Oral Probiotic. J. Agric. Food Chem..

[133] Li L., Li J., Wang Y., Liu X., Li S., Wu Y., Tang W., Qiu Y. (2021). Resveratrol Prevents Inflammation and Oxidative Stress Response in LPS-Induced Human Gingival Fibroblasts by Targeting the PI3K/AKT and Wnt/β-Catenin Signaling Pathways. Genet. Mol. Biol..

[134] Shimazu K., Ookoshi K., Fukumitsu S., Kagami H., Mitsuhata C., Nomura R., Aida K. (2024). Effects of Oleanolic Acid Derived from Wine Pomace on Periodontopathic Bacterial Growth in Healthy Individuals: A Randomized Placebo-Controlled Study. Dent. J..

[135] Gosset-Erard C., Zhao M., Lordel-Madeleine S., Ennahar S. (2021). Identification of Punicalagin as the Bioactive Compound behind the Antimicrobial Activity of Pomegranate (*Punica granatum* L.) Peels. Food Chem..

[136] Čolić M., Miljuš N., Đokić J., Bekić M., Krivokuća A., Tomić S., Radojević D., Radanović M., Eraković M., Ismaili B. (2023). Pomegranate Peel Extract Differently Modulates Gene Expression in Gingiva-Derived Mesenchymal Stromal Cells under Physiological and Inflammatory Conditions. Int. J. Mol. Sci..

[137] Sengun M.C., Gunpinar S. (2021). Effects of Systemic Hydroxytyrosol Application in Experimental Periodontitis of Rats. Int. J. Plant Based Pharm..

[138] Ramos A.C., de Melo M.C.F., de Lima A.K.M., Souza T.G., Ferreira A.K.A., de Macêdo T.S., de França Caldas Júnior A., da Silva Telles A.M., Godoy G.P. (2025). Biosafety and Protective Effects of Tyrosol from *Olea europaea* L. in Gingivitis: In Vitro and in Vivo Studies. Pesqui. Bras. Odontopediatria Clin. Integr..

[139] Adampour Z., Yilmaz Özturk B., Yenice Gursu B., Dag İ. (2025). In Vitro Antibiofilm Activity of Tyrosol against Single and Dual-Species Biofilms of Candida Tropicalis and Streptococcus Mutans. Turk. J. Biol..

[140] Gugliandolo E., Fusco R., D’Amico R., Peditto M., Oteri G., Di Paola R., Cuzzocrea S., Navarra M. (2019). Treatment with a Flavonoid-Rich Fraction of Bergamot Juice Improved Lipopolysaccharide-Induced Periodontitis in Rats. Front. Pharmacol..

[141] Yue J., Yang H., Liu S., Song F., Guo J., Huang C. (2018). Influence of Naringenin on the Biofilm Formation of *Streptococcus mutans*. J. Dent..

[142] Kong C., Zhang H., Li L., Liu Z. (2022). Effects of Green Tea Extract Epigallocatechin-3-Gallate (EGCG) on Oral Disease-Associated Microbes: A Review. J. Oral Microbiol..

[143] Malcangi G., Patano A., Ciocia A.M., Netti A., Viapiano F., Palumbo I., Trilli I., Guglielmo M., Inchingolo A.D., Dipalma G. (2023). Benefits of Natural Antioxidants on Oral Health. Antioxidants.

[144] Kibriya S., Srinivasan I., Setty J.V., Anu S., Khan B.S. (2023). Characterization of Cocoa Bean Husk Extract Particles and Its Comparison as a Mouthrinse with Different Vehicles in Children Aged 7–12 Years. Int. J. Clin. Pediatr. Dent..

[145] Viglianisi G., Polizzi A., Grippaudo C., Cocuzza S., Leonardi R., Isola G. (2024). Chemopreventive and Biological Strategies in the Management of Oral Potentially Malignant and Malignant Disorders. Bioengineering.

[146] Christine D.W. (2009). Grape Products and Oral Health. J. Nutr..

[147] Lin X., Zhu L., He J. (2022). Morphogenesis, Growth Cycle and Molecular Regulation of Hair Follicles. Front. Cell Dev. Biol..

[148] Zhao J., Li H., Zhou R., Ma G., Dekker J.D., Tucker H.O., Yao Z., Guo X. (2015). Foxp1 Regulates the Proliferation of Hair Follicle Stem Cells in Response to Oxidative Stress during Hair Cycling. PLoS ONE.

[149] Du F., Li J., Zhang S., Zeng X., Nie J., Li Z. (2024). Oxidative Stress in Hair Follicle Development and Hair Growth: Signalling Pathways, Intervening Mechanisms and Potential of Natural Antioxidants. J. Cell. Mol. Med..

[150] Mustafa A.I., Khashaba R.A., Fawzy E., Baghdady S.M.A., Rezk S.M. (2021). Cross Talk between Oxidative Stress and Inflammation in Alopecia Areata. J. Cosmet. Dermatol..

[151] Trüeb R.M. (2021). Oxidative Stress and Its Impact on Skin, Scalp and Hair. Int. J. Cosmet. Sci..

[152] Le Floc’h C., Cheniti A., Connétable S., Piccardi N., Vincenzi C., Tosti A. (2015). Effect of a Nutritional Supplement on Hair Loss in Women. J. Cosmet. Dermatol..

[153] Katoulis A.C., Liakou A.I., Koumaki D., Vakirlis E., Tsantes A.G., Mortaki D., Bozi E., Ioannides D. (2020). A Randomized, Single-blinded, Vehicle-controlled Study of a Topical Active Blend in the Treatment of Androgenetic Alopecia. Dermatol. Ther..

[154] Echeverria M.C., Nuti M. (2017). Valorisation of the Residues of Coffee Agro-Industry: Perspectives and Limitations. Open Waste Manag. J..

[155] Hoseini M., Cocco S., Casucci C., Cardelli V., Corti G. (2021). Coffee By-Products Derived Resources. A Review. Biomass Bioenergy.

[156] Muangsanguan A., Linsaenkart P., Chaitep T., Sangta J., Sommano S.R., Sringarm K., Arjin C., Rachtanapun P., Jantanasakulwong K., Phimolsiripol Y. (2023). Hair Growth Promotion and Anti-Hair Loss Effects of By-Products Arabica Coffee Pulp Extracts Using Supercritical Fluid Extraction. Foods.

[157] Wisetkomolmat J., Arjin C., Satsook A., Seel-audom M., Ruksiriwanich W., Prom-u-Thai C., Sringarm K. (2022). Comparative Analysis of Nutritional Components and Phytochemical Attributes of Selected Thai Rice Bran. Front. Nutr..

[158] Muangsanguan A., Ruksiriwanich W., Arjin C., Jamjod S., Prom-u-Thai C., Jantrawut P., Rachtanapun P., Hnorkaew P., Satsook A., Sainakham M. (2024). Comparison of In Vitro Hair Growth Promotion and Anti-Hair Loss Potential of Thai Rice By-Product from Oryza Sativa L. Cv. Buebang 3 CMU and Sanpatong. Plants.

[159] You J., Woo J., Roh K., Ryu D., Jang Y., Cho E., Park D., Jung E. (2023). Assessment of the Anti-hair Loss Potential of *Camellia japonica* Fruit Shell Extract in Vitro. Int. J. Cosmet. Sci..

[160] Ma L., Shen H., Fang C., Chen T., Wang J. (2022). Camellia Seed Cake Extract Supports Hair Growth by Abrogating the Effect of Dihydrotestosterone in Cultured Human Dermal Papilla Cells. Molecules.

[161] Wang J., Shen H., Chen T., Ma L. (2022). Hair Growth-promoting Effects of Camellia Seed Cake Extract in Human Dermal Papilla Cells and C57BL/6 Mice. J. Cosmet. Dermatol..

[162] Chauhan S., Pandit N.K., Mohanty A., Meena S.S. (2025). Resource Recovery of Bioactive Compounds from Food Waste and Their Diverse Industrial Applications. Biomass Convers. Biorefin..

[163] Rodrigues R., Oliveira M.B.P.P., Alves R.C. (2023). Chlorogenic Acids and Caffeine from Coffee By-Products: A Review on Skincare Applications. Cosmetics.

[164] Grigolon G., Nowak K., Poigny S., Hubert J., Kotland A., Waldschütz L., Wandrey F. (2023). From Coffee Waste to Active Ingredient for Cosmetic Applications. Int. J. Mol. Sci..

[165] Fernandes G.L., Vieira A.P.M., Danelon M., Emerenciano N.G., Berretta A.A., Buszinski A.F.M., Hori J.I., Lima M.H.F.d., Reis T.F.d., Lima J.A.d. (2022). Pomegranate Extract Potentiates the Anti-Demineralizing, Anti-Biofilm, and Anti-Inflammatory Actions of Non-Alcoholic Mouthwash When Associated with Sodium-Fluoride Trimetaphosphate. Antibiotics.

[166] Aït-Kaddour A., Hassoun A., Tarchi I., Loudiyi M., Boukria O., Cahyana Y., Ozogul F., Khwaldia K. (2024). Transforming Plant-Based Waste and by-Products into Valuable Products Using Various “Food Industry 4.0” Enabling Technologies: A Literature Review. Sci. Total Environ..

[167] Casanova F., Santos L. (2016). Encapsulation of Cosmetic Active Ingredients for Topical Application—A Review. J. Microencapsul..

[168] Bouizgma K., Rabbah N., Abbas Z., Abourriche A. (2025). Unlocking Sustainable Extraction of Natural Antioxidants: Green Solvents, Smart Technologies, Scalability and Future Directions. Sep. Sci. Technol..

[169] Thakali A., MacRae J.D., Isenhour C., Blackmer T. (2022). Composition and Contamination of Source Separated Food Waste from Different Sources and Regulatory Environments. J. Environ. Manag..

[170] Socas-Rodríguez B., Álvarez-Rivera G., Valdés A., Ibáñez E., Cifuentes A. (2021). Food By-Products and Food Wastes: Are They Safe Enough for Their Valorization?. Trends Food Sci. Technol..

[171] Ferreira M., Matos A., Couras A., Marto J., Ribeiro H. (2022). Overview of Cosmetic Regulatory Frameworks around the World. Cosmetics.

[172] Monika, Pragi, Kumar V., Jyoti, Sarswa A., Shrikant, Malik G., Kumari S. (2025). The Global Cosmetic Industry: Regulatory Challenges and Innovations for Ensuring Safety and Compliance. Curr. Cosmet. Sci..

